# Supplementary material for: Patterns of SARS-CoV-2 seropositivity among essential workers in long term care and retirement homes in Ontario, Canada: A descriptive cross-sectional study
Source: PLOS Glob Public Health. 2025 Mar 28;5(3):e0004294. doi: 10.1371/journal.pgph.0004294 (PMC11952236; doi:10.1371/journal.pgph.0004294)
Supplement: S1 Text — (DOCX) [file pgph.0004294.s001.docx]

Project partners, included but were not limited to, Family Councils Ontario, Ontario LTC Association, Local Health Integration Networks, The Toronto Region COVID-19 Long-term care/ Congregate Care Table, Ontario Personal Support Worker Association, Toronto and Ottawa Public Health Units, Healthcare Excellence Canada, Public Health Ontario, Regional Geriatric Program of Ontario, and academic institutions in Ontario (Unity Health Toronto, McMaster University, University of Toronto, The Ottawa Hospital, Sinai Health, Toronto Metropolitan University).

For a full list of study partners please visit: wellness-hub.ca
