# Supplementary material for: Patterns of SARS-CoV-2 seropositivity among essential workers in long term care and retirement homes in Ontario, Canada: A descriptive cross-sectional study
Source: PLOS Glob Public Health. 2025 Mar 28;5(3):e0004294. doi: 10.1371/journal.pgph.0004294 (PMC11952236; doi:10.1371/journal.pgph.0004294)
Supplement: S2 Text — (DOCX) [file pgph.0004294.s002.docx]

**Wellness Hub Demographic Questionnaire**

**(LTCH/RH Staff)**

Thank you very much for your participation in the Wellness Hub research study. As a part of your participation in this research study, we are looking to collect demographic information about you. This information will be used to better understand factors associated with SARS-CoV-2 infection (i.e., COVID-19).

**This survey should take no more than 20-25 minutes to complete.**

When you are finished the questionnaire, please return to a Wellness Hub team member and/or your Long Term Care Home (**LTCH**)/ Retirement Home (**RH**). Please note that continued consent to participate in this component of the Wellness Hub research study is indicated by proceeding with the survey.

**Survey Questions**

For this survey, a **“household”** refers to everyone who may share a kitchen and living spaces (e.g., living room, bathroom) in the same place you live, and who sleeps at least one night per week at the same home as you **for at least 4 weeks**. If you live in more than one household (that is, you sleep in more than one home in a given week), please combine the two households together for the questions about your household(s).

| **Questions** | | |
| --- | --- | --- |
| 1. **Please enter your participant ID.** | | |
| **____ ____ ____ ____ ____ ____ ____ ____** | | |
| 1. **Please enter the date that you are completing this questionnaire.** | | |
| **____ ____ / ____ ____ / ____ ____ (DD/ MM/ YY)** | | |
| 1. **What is your age in Years?** | | |
| **____ ____ ____ (Years)** (e.g., 45 years old) | | |
| 1. **What is your employment status in this LTCH/RH?** Select all that apply: | | |
| Full-time permanent | Full-time temporary | Agency or contract work |
| Part-time permanent | Part-time temporary | Prefer not to answer |
| Other:  ___________________________________________________________________ | | |

| 1. **What language are you most comfortable speaking?**   Select all that apply: | | | | | | |
| --- | --- | --- | --- | --- | --- | --- |
| English | French | | Arabic | | Bengali | Cantonese |
| Vietnamese | Farsi (Persian) | | German | | Greek | Gujarati |
| Italian | Korean | | Mandarin | | Polish | Portuguese |
| Punjabi | Russian | | Spanish | | Somali | Tagalog |
| Tamil | Urdu | | Chinese  (not Cantonese / Mandarin) | | Other:  ______________________________ | |
| 1. **What was your total income before taxes last year, from just your work/employment?**   Choose one: | | | | | | |
| $0 - $29,999 | | $90,000 - $119,999 | | I do not know | | |
| $30,000 - $59,999 | | $120,000 - $149,999 | | Prefer not to answer | | |
| $60,000 - $89,999 | | $150,000 or more | |  | | |
| 1. **What was your total household income before taxes last year?**   Choose one: | | | | | | |
| $0 - $29,999 | | $90,000 - $119,999 | | I do not know | | |
| $30,000 - $59,999 | | $120,000 - $149,999 | | Prefer not to answer | | |
| $60,000 - $89,999 | | $150,000 or more | |  | | |
| 1. **What was your assigned sex at birth?** Choose one: | | | | | | |
| Male | | Female | | Prefer not to answer | | |
| Prefer to self-describe:  _________________________________________________________ | | | | | | |
| 1. **What gender do you currently most identify with?** Choose one: | | | | | | |
| Man | | Woman | | Two-spirit | | |
| Non-binary, gender queer, gender fluid, agender, or a similar identity | | Prefer not to answer | | Prefer to self-describe:  ________________________ | | |

| 1. **How would you describe your ethnicity or race?** Select all that apply: | | |
| --- | --- | --- |
| Black – African | East Asian – Korean | Middle Eastern – Middle Eastern/ West Asian |
| Black – Afro-Caribbean | East Asian – Mongolian, Taiwanese, and others | Southeast Asian – Cambodian, Indonesian, Laotian, Vietnamese, and others |
| Black – North American | LatinX or Hispanic – Caribbean | Southeast Asian – Filipino |
| Black – South and Central American | LatinX or Hispanic – Central American | Southeast Asian – East Indian, Pakistani, Sri Lankan, and others |
| Black – Afro-European | LatinX or Hispanic – European | West Asian – Afghan, Iranian, Turkish, and others |
| Central Asian – Kazakhstani, Uzbekistani, and others | LatinX or Hispanic – South American | White – European |
| East Asian – Chinese | Middle Eastern – North African | White – North American |
| East Asian – Japanese | Prefer to self describe: | Prefer not to answer |
| Other:  _______________________ | _________________________ |  |

| 1. **What is the highest level of education you have completed?** Choose one: | | |
| --- | --- | --- |
| Elementary School | University bachelor’s  degree | Trade certificate, Vocational  School or Apprenticeship  training |
| Some high school | University graduate degree  (e.g., Master’s, Doctorate) | Completed professional  school |
| High school graduation | Completed professional  school (e.g., nursing school,  Doctor of Medicine, Juris  Doctor) | Non-university certificate or  diploma from a community  college, CEGEP |
| Other:  ________________________________________________ | | Prefer not to answer |

| **12a. Did you get the flu shot in 2020?** Choose one: | | |
| --- | --- | --- |
| Yes | No | I do not know |
| **12b. Did you get the flu shot in 2021?** Choose one: | | |
| Yes | No | I do not know |
| **12c. Did you get the flu shot in 2022?** Choose one: | | |
| Yes | No | I do not know |

| **Supplemental questions if you do NOT consent to linking your**  **Ontario Health (OHIP) Card to your study data.**  **If you have provided your OHIP card to the Wellness Hub team,**  **do not complete question 13.** | | | | | |
| --- | --- | --- | --- | --- | --- |
| **13a. Were you ever admitted to hospital with COVID-19?** Choose one: | | | | | |
| **Yes**  Yes | | **No** | |  | |
| **13b. Have you ever been tested for COVID-19 by nasal/throat swab?** Choose one: | | | | | |
| **Yes** | | **No** | | **I am not sure /**  **I do not remember** | |
| **13c. How many times were you tested since the start of the pandemic (~March 2020) and Now (i.e., date completing questionnaire)?** Choose one: | | | | | |
| **0** | **1** | **2** | **3** | **4** | **5 and over** |
| **13d. Have you ever tested positive for COVID-19?** Choose one: | | | | | |
| **Yes** | | **No** | | **I am not sure /**  **I do not remember** | |
| **13e. If you have previously tested positive for COVID-19, when was your first positive test** | | | | | |
| **___ ___/___ ___/___ ___ (DD/MM/YY)** Put an estimate [e.g., Early/Mid/Late month]  if you do not remember  Not Applicable | | | | | |
| **13f. If you have previously tested positive for COVID-19, when was your second positive test** | | | | | |
| **___ ___/___ ___/___ ___ (DD/MM/YY)** Put an estimate [e.g., Early/Mid/Late month]  if you do not remember  Not Applicable | | | | | |
| **13g. If you have previously tested positive for COVID-19, when was your third positive test** | | | | | |
| **___ ___/___ ___/___ ___ (DD/MM/YY)** Put an estimate [e.g., Early/Mid/Late month]  if you do not remember  Not Applicable | | | | | |
| **13h. If you have previously tested positive for COVID-19, when was your fourth positive test** | | | | | |
| **___ ___/___ ___/___ ___ (DD/MM/YY)** Put an estimate [e.g., Early/Mid/Late month]  if you do not remember  Not Applicable | | | | | |
| **13i. If you have previously tested positive for COVID-19, when was your fifth positive test** | | | | | |
| **___ ___/___ ___/___ ___ (DD/MM/YY)** Put an estimate [e.g., Early/Mid/Late month]  if you do not remember  Not Applicable | | | | | |
| **13j. If you have previously tested positive for COVID-19; When you tested positive, did you miss work?** Choose one: | | | | | |
| **Yes** | | **No** | | **I am not sure /**  **I do not remember** | |
| **If yes, how many days of work did you miss**? ___ ___ [Number] | | | | | |
| Not applicable, I did not test positive for COVID-19 | | | | | |

| **COVID-19 Vaccine Information** | | | | | | | | | | | | | |
| --- | --- | --- | --- | --- | --- | --- | --- | --- | --- | --- | --- | --- | --- |
| **14. Have you been vaccinated against COVID-19?** Choose one:  *Answer ‘Yes’ if you have received at least one dose of the COVID-19 vaccine.*    *Note: Certain types of vaccines require more than one dose to protect against COVID-19. You would have been informed at the time of vaccination if you needed (an) additional dose(s).* | | | | | | | | | | | | | |
| Yes | No | | | **If No, do you have an appointment booked to be vaccinated?** | | | | | | | | | |
|  |  | | | | | | | Yes | | No | | | |
| **14a. If you have been vaccinated, How many doses of the COVID-19 vaccine have you received so far?** Choose one: | | | | | | | | | | | | | |
| One dose | | | | | Two doses | | | | | | | Three doses | |
| Four dose | | | | | Five doses | | | | | | | Six doses | |
| **The next series of questions applies if you checked ‘Yes’ to being vaccinated OR if you have an appointment booked to be vaccinated.**  **If this does not apply, go to question 14t.** | | | | | | | | | | | | | |
| **14b. If you have received your first dose or have an appointment booked to receive your first dose, When did (or when will) you receive your first dose of the COVID-19 vaccine?** | | | | | | | | | | | | | |
| **___ ___/___ ___/___ ___ (DD/MM/YY)** | | | | | | | | | | | | | |
| *(If you received your first dose)* **If you do not remember the exact date, did you receive the vaccine:** | | | | | | | | | | | | | |
| A few days ago | | | A week ago | | | | | | A few weeks ago | | | |  |
| More than 1 month ago | | | More than 6 months ago | | | | | | I do not know / I do not remember | | | |  |
| Other:  __________________________________________________ | | | | | | | | | | | | |  |
| **14c. If you have received your first dose or have an appointment booked to receive your first dose, Which vaccine did (or when will) you receive for your first dose?** Choose one: | | | | | | | | | | | | | |
| Pfizer and  BioNTech mRNA | | AstraZeneca  Oxford | | | | | Other:  _______________________________ | | | | | | |
| Moderna mRNA | | Janssen | | | | | I am not sure / I do not remember | | | | | | |
| **14d. If you have received your first dose or have an appointment booked to receive your first dose, Where did (or will you) you receive your first dose?** Choose one: | | | | | | | | | | | | | |
| I travelled/will travel to a clinic that was vaccinating | | | | I travelled/will travel to a pharmacy that was vaccinating | | | | | | | I received/will receive the vaccine at my place of work | | |
| I travelled/will travel to a hospital that was vaccinating | | | | I received/will receive the vaccine at my family doctor’s office | | | | | | | I received/will receive the vaccine at my LTCH/RH **where I reside** | | |
| I am not sure / I do not remember | | | | | | Other:  ______________________________________ | | | | | | | |

| **14e. If you have received your second dose or have an appointment booked to receive your second dose, When did (or when will) you receive your second dose of the COVID-19 vaccine?** | | | | | | | | |
| --- | --- | --- | --- | --- | --- | --- | --- | --- |
| **___ ___/___ ___/___ ___ (DD/MM/YY)** | | | | | | | | |
| *(If you received your second dose)* **If you do not remember the exact date, did you receive the vaccine:** | | | | | | | | |
| A few days ago | | A week ago | | | | A few weeks ago | |  |
| More than 1 month ago | | More than 6 months ago | | | | I do not know / I do not remember | |  |
| Other:  __________________________________________________ | | | | | | | |  |
| **14f. If you have received your second dose or have an appointment booked to receive your second dose, Which vaccine did (or when will) you receive for your second dose?**  Choose one: | | | | | | | | |
| Pfizer and  BioNTech mRNA | AstraZeneca  Oxford | | | | Other:  _______________________________ | | | |
| Moderna mRNA | Janssen | | | | I am not sure / I do not remember | | | |
| **14g. If you have received your second dose or have an appointment booked to receive your second dose, Where did (or will you) you receive your second dose?**  Choose one: | | | | | | | | |
| I travelled/will travel to a clinic that was vaccinating | | | I travelled/will travel to a pharmacy that was vaccinating | | | | I received/will receive the vaccine at my place of work | |
| I travelled/will travel to a hospital that was vaccinating | | | I received/will receive the vaccine at my family doctor’s office | | | | I received/will receive the vaccine at my LTCH/RH **where I reside** | |
| I am not sure / I do not remember | | | | Other:  ______________________________________ | | | | |

| **14h. If you have received your third dose or have an appointment booked to receive your third dose, When did (or when will) you receive your third dose of the COVID-19 vaccine?** | | | | | | | | |
| --- | --- | --- | --- | --- | --- | --- | --- | --- |
| **___ ___/___ ___/___ ___ (DD/MM/YY)** | | | | | | | | |
| *(If you received your third dose)* **If you do not remember the exact date, did you receive the vaccine:** | | | | | | | | |
| A few days ago | | A week ago | | | | A few weeks ago | |  |
| More than 1 month ago | | More than 6 months ago | | | | I do not know / I do not remember | |  |
| Other:  __________________________________________________ | | | | | | | |  |
| **14i. If you have received your third dose or have an appointment booked to receive your third dose, Which vaccine did (or when will) you receive for your third dose?**  Choose one: | | | | | | | | |
| Pfizer and  BioNTech mRNA | AstraZeneca  Oxford | | | | Other:  _______________________________ | | | |
| Moderna mRNA | Janssen | | | | I am not sure / I do not remember | | | |
| **14j. If you have received your third dose or have an appointment booked to receive your third dose, Where did (or will you) you receive your third dose?** Choose one: | | | | | | | | |
| I travelled/will travel to a clinic that was vaccinating | | | I travelled/will travel to a pharmacy that was vaccinating | | | | I received/will receive the vaccine at my place of work | |
| I travelled/will travel to a hospital that was vaccinating | | | I received/will receive the vaccine at my family doctor’s office | | | | I received/will receive the vaccine at my LTCH/RH **where I reside** | |
| I am not sure / I do not remember | | | | Other:  ______________________________________ | | | | |

| **14k. If you have received your fourth dose or have an appointment booked to receive your fourth dose, When did (or when will) you receive your fourth dose of the COVID-19 vaccine?** | | | | | | | | |
| --- | --- | --- | --- | --- | --- | --- | --- | --- |
| **___ ___/___ ___/___ ___ (DD/MM/YY)** | | | | | | | | |
| *(If you received your fourth dose)* **If you do not remember the exact date, did you receive the vaccine:** | | | | | | | | |
| A few days ago | | A week ago | | | | A few weeks ago | |  |
| More than 1 month ago | | More than 6 months ago | | | | I do not know / I do not remember | |  |
| Other:  ____________________________________________________________ | | | | | | | |  |
| **14l. If you have received your fourth dose or have an appointment booked to receive your fourth dose, Which vaccine did (or when will) you receive for your fourth dose?**  Choose one: | | | | | | | | |
| Pfizer and  BioNTech mRNA | AstraZeneca  Oxford | | | | Other:  _______________________________ | | | |
| Moderna mRNA | Janssen | | | | I am not sure / I do not remember | | | |
| **14m. If you have received your fourth dose or have an appointment booked to receive your fourth dose, Where did (or will you) you receive your fourth dose?** Choose one: | | | | | | | | |
| I travelled/will travel to a clinic that was vaccinating | | | I travelled/will travel to a pharmacy that was vaccinating | | | | I received/will receive the vaccine at my place of work | |
| I travelled/will travel to a hospital that was vaccinating | | | I received/will receive the vaccine at my family doctor’s office | | | | I received/will receive the vaccine at my LTCH/RH **where I reside** | |
| I am not sure / I do not remember | | | | Other:  ______________________________________ | | | | |

| **14n. If you have received your fifth dose or have an appointment booked to receive your fifth dose, When did (or when will) you receive your fifth dose of the COVID-19 vaccine?** | | | | | | | | |
| --- | --- | --- | --- | --- | --- | --- | --- | --- |
| **___ ___/___ ___/___ ___ (DD/MM/YY)** | | | | | | | | |
| *(If you received your fifth dose)* **If you do not remember the exact date, did you receive the vaccine:** | | | | | | | | |
| A few days ago | | A week ago | | | | A few weeks ago | |  |
| More than 1 month ago | | More than 6 months ago | | | | I do not know / I do not remember | |  |
| Other:  __________________________________________________ | | | | | | | |  |
| **14o. If you have received your fifth dose or have an appointment booked to receive your fifth dose, Which vaccine did (or when will) you receive for your fifth dose?**  Choose one: | | | | | | | | |
| Pfizer and  BioNTech mRNA | AstraZeneca  Oxford | | | | Other:  _______________________________ | | | |
| Moderna mRNA | Janssen | | | | I am not sure / I do not remember | | | |
| **14p. If you have received your fifth dose or have an appointment booked to receive your fifth dose, Where did (or will you) you receive your fifth dose?** Choose one: | | | | | | | | |
| I travelled/will travel to a clinic that was vaccinating | | | I travelled/will travel to a pharmacy that was vaccinating | | | | I received/will receive the vaccine at my place of work | |
| I travelled/will travel to a hospital that was vaccinating | | | I received/will receive the vaccine at my family doctor’s office | | | | I received/will receive the vaccine at my LTCH/RH **where I reside** | |
| I am not sure / I do not remember | | | | Other:  ______________________________________ | | | | |

| **14q. If you have received your sixth dose or have an appointment booked to receive your sixth dose, When did (or when will) you receive your sixth dose of the COVID-19 vaccine?** | | | | | | | | |
| --- | --- | --- | --- | --- | --- | --- | --- | --- |
| **___ ___/___ ___/___ ___ (DD/MM/YY)** | | | | | | | | |
| *(If you received your sixth dose)* **If you do not remember the exact date, did you receive the vaccine:** | | | | | | | | |
| A few days ago | | A week ago | | | | A few weeks ago | |  |
| More than 1 month ago | | More than 6 months ago | | | | I do not know / I do not remember | |  |
| Other:  __________________________________________________ | | | | | | | |  |
| **14r. If you have received your sixth dose or have an appointment booked to receive your sixth dose, Which vaccine did (or when will) you receive for your sixth dose?**  Choose one: | | | | | | | | |
| Pfizer and  BioNTech mRNA | AstraZeneca  Oxford | | | | Other:  _______________________________ | | | |
| Moderna mRNA | Janssen | | | | I am not sure / I do not remember | | | |
| **14s. If you have received your sixth dose or have an appointment booked to receive your sixth dose, Where did (or will you) you receive your sixth dose?** Choose one: | | | | | | | | |
| I travelled/will travel to a clinic that was vaccinating | | | I travelled/will travel to a pharmacy that was vaccinating | | | | I received/will receive the vaccine at my place of work | |
| I travelled/will travel to a hospital that was vaccinating | | | I received/will receive the vaccine at my family doctor’s office | | | | I received/will receive the vaccine at my LTCH/RH **where I reside** | |
| I am not sure / I do not remember | | | | Other:  ______________________________________ | | | | |

| **The next series applies if you checked ‘NO’ to being vaccinated AND DO NOT have an appointment booked to be vaccinated.** | | | |
| --- | --- | --- | --- |
| **14t. If you have NOT been vaccinated or do NOT have an appointment booked to be vaccinated, Why did you not receive a COVID-19 vaccine?** Choose one: | | | |
| I was not offered a vaccine | | I was not eligible for a vaccine (e.g., presence of a health condition) | |
| I did not want a vaccine. If so why:  __________________________________ | | Other:  ____________________________________ | |
| **14u. If you have NOT been vaccinated or do NOT have an appointment booked to be vaccinated, Do you intend to be vaccinated against COVID-19?** Choose one: | | | |
| Yes | No | | Unsure |

| **Occupational Health and Safety Questions**  **Please note that these questions apply to your job title at your primary LTCH/RH, not to any additional jobs you may have.** | | | | | |
| --- | --- | --- | --- | --- | --- |
| **15. What best describes your job title at this LTCH/RH?** Select **all** that apply: | | | | | |
| Nurse | Laundry staff | | Reception | | |
| Personal support worker | Kitchen staff | | Administration | | |
| Physician | Custodial /  Housekeeping staff | | Other:  ________________________ | | |
| **16. Do you belong to a union?** Choose one: | | | | | |
| Yes. If yes, please list which union(s):  _______________________________________ | | No | | | I do not know |
| **17. Is there a Joint Health and Safety Committee in your workplace?** Choose one: | | | | | |
| Yes | No | | | I do not know | |
| **18. Have you received general health and safety training at your workplace? (e.g., explaining the Occupational Health and Safety Act, the right to know, participate and refuse unsafe work).** Choose one: | | | | | |
| Yes | No | | | I do not know | |
| **19. Have you had Workplace Hazardous Materials Information System (WHMIS) training at your workplace?** Choose one: | | | | | |
| Yes | No | | | I do not know | |
| **20. Have you received Infection Prevention and Control (IPAC) training at your workplace?** Choose one: | | | | | |
| Yes | No | | | I do not know | |
| **If yes, please check if you have received training on the following:** | | | | | |
| How to use gloves | | | | | |
| How to use facial protection (mask, respirator, goggles, safety shield) | | | | | |

| **Household and Occupation Questions** | | | | |
| --- | --- | --- | --- | --- |
| **21. Please enter your postal code of where you primarily reside/live (sleep most nights of the week).** This information will be used to explore relationships between neighbourhood factors and COVID-19 outcomes. | | | | |
| **_____ _____ _____ _____ _____ _____** | | | | |
| **22. In addition to your position at the LTCH/RH, have you worked in any other health-care settings since the start of the pandemic (~March 2020)?** Select **all** that apply: | | | | |
| Yes, other LTCH | Yes, other RH | | Yes, hospital or clinic | |
| Yes, shelter, group home or other congregate care setting | Yes, home care worker/services | | Yes, other healthcare facility.  Describe:  _________________________________ | |
| No | | | | |
| **If yes to any of the above, have you been working in this/these other health-care setting(s) in the last month?** | | | | |
| Yes | | No | |  |

| **23. If you have worked in another health-care setting since the start of the pandemic (~March 2020) was there ever a COVID outbreak at this/these other health-care setting(s) while you were working there?** Choose one: | | |
| --- | --- | --- |
| Yes | No | I do not know |
| **24. In addition to your position at this LTCH/RH, have you worked outside of your home in any non-health-care settings since the start of the pandemic (~March 2020)? (Working can refer to any employment, jobs, and occupations).** Choose one: | | |
| No. I have not worked in any non-health-care settings since the start of the pandemic (~March 2020) | No. I have worked in a non-health-care setting since the start of the pandemic (~March 2020) but I was able to do most of my work remotely (e.g. I worked from home) | Yes, I have been an ‘essential’ or ‘frontline’ worker *(job where you were NOT able to spend the majority of your working hours working remotely or from home)* in a non-health-care setting since the start of the pandemic (~March 2020) |
| **If yes to the above, have you worked as an ‘essential’ or ‘frontline’ worker in this  non-health-care setting in the last month?** | | |
| Yes | No |  |
| **25. If you have worked outside of your home in a non-health-care setting since the start of the pandemic (~March 2020) was there ever a COVID outbreak at this other health-care setting while you were working there?** Choose one: | | |
| Yes | No | I do not know |
| **26. Were you attending in-person school, education, and/or training anytime between March 2020 and now?** Choose one: | | |
| Yes | No |  |
| **If yes, are you currently attending in person school, education, and/or training?** | | |
| Yes | No |  |

| **27. If applicable, in the previous month, how did you get to and from your place(s) of work on most days?** Select all that apply: | | | | | | | | | |
| --- | --- | --- | --- | --- | --- | --- | --- | --- | --- |
| **m** Not applicable, I work from home | | | | | Drive with another member of my household | | | | Public  transport |
| Not applicable, I am not working | | | | | Drive alone | | | | Taxi |
| Carpool or ride-share with at least one person from the same LTCH/ RH where I work | | | | | Carpool or ride-share with at least one other person outside my household | | | | Walk |
| Other:  ____________________________________________________ | | | | | | | | | |
| **28. Are you aware of financial supports available to workers if sick (e.g., Canada Recovery Sickness Benefit [CRSB])?** Choose one: | | | | | | | | | |
| Yes, I am aware and I have accessed these supports since the start of the pandemic (~March 2020) | | | Yes, I am aware but I have not accessed these supports since the start of the pandemic (~March 2020) | | | | No, I am not aware of these supports | | |
| **29. Did you have paid sick leave through your LTCH/RH since the start of the pandemic (~March 2020) and now?** Choose one: | | | | | | | | | |
|  | Yes | No | | | | I do not know | | | |
| **If yes, how many days of paid sick leave do you have?** _____________ [days] | | | | | | | | | |
| **If yes, did you have paid sick leave before the province program of 3 days (announced**  **May 2021)?** | | | | | | | | | |
|  | Yes | No | | | | I do not know | | | |
|  | | | | | | | | | |
| **30. If applicable (if Y to Q22 and/or Q24), did you have paid sick leave through any of your additional places of work since the start of the pandemic (~March 2020) and now?** Choose one | | | | | | | | | |
| Yes | | | | No | | | | I do not know | |
| Not applicable, I do not have an additional place of work | | | | | | | | | |
| **If yes, how many days of paid sick leave do you have?** _____________ [days] | | | | | | | | | |
|  | | | | | | | | | |
| **If yes, did you have paid sick leave before the province program of 3 days (announced May 2021)?** | | | | | | | | | |
| Yes | | | | No | | | | I do not know | |

| **31. How many people currently (i.e., within the last 4 weeks) live in your household (including yourself)?**  *Please note that a “household” refers to everyone who may share a kitchen and living spaces (e.g., living room, bathroom) in the same place you live, and who sleeps at least one night per week at the same home as you for at least 4 weeks. If you live in more than one household (that is, you sleep in more than one home in a given week), please combine the two households together for the questions about your household(s).* |
| --- |
| **___ ___ [Number]** |
| **32. What is the total number of unique household members that have lived in your household since the start of the pandemic (~March 2020) and now (including yourself)?**  *Please note that a “household” refers to everyone who may share a kitchen and living spaces (e.g., living room, bathroom) in the same place you live, and who sleeps at least one night per week at the same home as you for at least 4 weeks. If you live in more than one household (that is, you sleep in more than one home in a given week), please combine the two households together for the questions about your household(s).* |
| **___ ___ [Number]** |

| **33. What best describes the type of housing you live in?** Choose one: | | |
| --- | --- | --- |
| House | Apartment/ condo (less than or equal to 5 stories) | Apartment/condo (more than 5 stories) |
| Basement apartment in house with separate entrance | Dormitory | Group home |
| Correctional facility | Shelter/ Hostel | Homeless/On street |
| Other:    ____________________________________________ | | Prefer not to answer |

| **The next series of questions applies if you checked that you live in a House, Apartment, Basement Apartment (i.e., not a shelter, hostel or group home).** |
| --- |
| **33a. How many bedrooms are in your household?** |
| **___ ___ [Number]** |
| **33b. How many bathrooms are in your household?** |
| **___ ___ [Number]** |

| **The next question applies if you checked that you live in a group home, dormitory, shelter/hostel, or correctional facility.** |
| --- |
| **35c. Approximately how many individuals reside in this residence (including yourself)?** |
| **___ ___ [Number]** |

| **The next series of questions applies if you checked that you live in a House, Apartment, Basement Apartment (i.e., not a shelter, hostel or group home).**  For EACH person who **currently (i.e., within the last 4 weeks)** lives in your household, please answer the following questions. For these questions**, you (i.e., the individual completing this questionnaire) are not counted as a household member.**  *Please note that a “household” refers to everyone who may share a kitchen and living spaces (e.g., living room, bathroom) in the same place you live, and who sleeps at least one night per week at the same home as you for at least 4 weeks. If you live in more than one household (that is, you sleep in more than one home in a given week), please combine the two households together for the questions about your household(s).* |
| --- |

| **Answer these Questions about Household Member 1**  **Please note: You (i.e., the individual completing this questionnaire) are not counted as a household member since you have answered these questions above. Please only answer these questions about additional members of your household.** | | | | | |
| --- | --- | --- | --- | --- | --- |
| **What is the age of this household member?**  **___ ___ ___ Years** (e.g., 45 years old) | | | | | |
| **Has this household member been living in your household over the past month?** | | | | | |
| Yes | | No | |  | |
| **Has this person ever been diagnosed with COVID-19?** | | | | | |
| Yes | | No | | I don’t know | |
| **If yes, what is their current outcome?** | | | | | |
| Isolating | In hospital | | Recovered | | Deceased |
| **Has this person received a COVID-19 vaccine?** Choose one: | | | | | |
| Yes, this person received 1 dose | Yes, this person received 2 doses | | Yes, this person received 3 doses | | Yes, this person received 4 doses |
| Yes, this person received 5 doses | Yes, this person received 6 doses | | No | | I do not know |
| **Has this household member worked outside the place of residence** (i.e., spent the majority of working hours NOT working from home/working remotely) **at some point since the start of the pandemic (~March 2020) and Now?** | | | | | |
| Not applicable | | No – worked mostly from home/remotely | | Yes | |
| **If yes, are they currently working outside of the place of residence (i.e., in the last month?)** | | | | | |
| Yes | | No | |  | |
| **Does this person currently attend in-person school, education, and/or training?**  Choose one: | | | | | |
| Yes | | No | | I do not know | |
|  | | | | | |

| **Answer these Questions about Household Member 2**  **Please note: You (i.e., the individual completing this questionnaire) are not counted as a household member since you have answered these questions above. Please only answer these questions about additional members of your household.** | | | | | |
| --- | --- | --- | --- | --- | --- |
| **What is the age of this household member?**  **___ ___ ___ Years** (e.g., 45 years old) | | | | | |
| **Has this household member been living in your household over the past month?** | | | | | |
| Yes | | No | |  | |
| **Has this person ever been diagnosed with COVID-19?** | | | | | |
| Yes | | No | | I don’t know | |
| **If yes, what is their current outcome?** | | | | | |
| Isolating | In hospital | | Recovered | | Deceased |
| **Has this person received a COVID-19 vaccine?** Choose one: | | | | | |
| Yes, this person received 1 dose | Yes, this person received 2 doses | | Yes, this person received 3 doses | | Yes, this person received 4 doses |
| Yes, this person received 5 doses | Yes, this person received 6 doses | | No | | I do not know |
| **Has this household member worked outside the place of residence** (i.e., spent the majority of working hours NOT working from home/working remotely) **at some point since the start of the pandemic (~March 2020) and Now?** | | | | | |
| Not applicable | | No – worked mostly from home/remotely | | Yes | |
| **If yes, are they currently working outside of the place of residence (i.e., in the last month?)** | | | | | |
| Yes | | No | |  | |
| **Does this person currently attend in-person school, education, and/or training?**  Choose one: | | | | | |
| Yes | | No | | I do not know | |
|  | | | | | |

| **Answer these Questions about Household Member 3**  **Please note: You (i.e., the individual completing this questionnaire) are not counted as a household member since you have answered these questions above. Please only answer these questions about additional members of your household.** | | | | | |
| --- | --- | --- | --- | --- | --- |
| **What is the age of this household member?**  **___ ___ ___ Years** (e.g., 45 years old) | | | | | |
| **Has this household member been living in your household over the past month?** | | | | | |
| Yes | | No | |  | |
| **Has this person ever been diagnosed with COVID-19?** | | | | | |
| Yes | | No | | I don’t know | |
| **If yes, what is their current outcome?** | | | | | |
| Isolating | In hospital | | Recovered | | Deceased |
| **Has this person received a COVID-19 vaccine?** Choose one: | | | | | |
| Yes, this person received 1 dose | Yes, this person received 2 doses | | Yes, this person received 3 doses | | Yes, this person received 4 doses |
| Yes, this person received 5 doses | Yes, this person received 6 doses | | No | | I do not know |
| **Has this household member worked outside the place of residence** (i.e., spent the majority of working hours NOT working from home/working remotely) **at some point since the start of the pandemic (~March 2020) and Now?** | | | | | |
| Not applicable | | No – worked mostly from home/remotely | | Yes | |
| **If yes, are they currently working outside of the place of residence (i.e., in the last month?)** | | | | | |
| Yes | | No | |  | |
| **Does this person currently attend in-person school, education, and/or training?**  Choose one: | | | | | |
| Yes | | No | | I do not know | |
| ***IF YOU HAVE ADDITIONAL HOUSEHOLD MEMBERS, PLEASE LET A WELLNESS HUB STAFF MEMBER KNOW AND THEY CAN PROVIDE YOU WITH ADDITIONAL BLANK HOUSEHOLD MEMBER FORMS** | | | | | |
