# Supplementary material for: Patterns of SARS-CoV-2 seropositivity among essential workers in long term care and retirement homes in Ontario, Canada: A descriptive cross-sectional study
Source: PLOS Glob Public Health. 2025 Mar 28;5(3):e0004294. doi: 10.1371/journal.pgph.0004294 (PMC11952236; doi:10.1371/journal.pgph.0004294)
Supplement: S3 Text — (DOCX) [file pgph.0004294.s003.docx]

**DBS interpretation**

Antibodies to SARS-CoV-2 were measured in an ELISA assay using three antigens: full-length spike trimer, its receptor-binding domain (RBD) and nucleocapsid (N) [1]. Seropositivity thresholds: spike (11.28 BAU/mL, 99% specificity), RBD (30.97 BAU/mL, 99% specificity) and N (34.46 BAU/mL, 99% specificity or 11.37 BAU/mL, 90% specificity).

*ELISA results:*

0/3 positive calls: no antibody response detected

1/3 positive calls: indeterminate

≥ 2/3 positive calls: positive for SARS-CoV-2 antibodies

As vaccines administered in Canada contain the spike protein, seropositivity to spike and RBD could be due to either vaccination or infection whereas antibodies to N would arise only from infection.

*Interpretation of positive ELISA results:*

spike + RBD (vaccination or past infection)

spike + N (past infection, may also be vaccinated)

RBD + N (past infection, may also be vaccinated)

Three positive calls (past infection, may also be vaccinated)

The sensitivity and specificity of the DBS samples were determined early in the pandemic based on an initial sample of 97 positives (confirmed COVID-19 infections) and 90 pre COVID-19 negative samples [1]; a seropositivity result requiring ≥ 2/3 antigens to be positive had a specificity of 100% and a sensitivity of 98%. Because seropositivity calls in vaccinated individuals rely on the N antigen and antibody levels to N are lower in vaccinated individuals [1-3], the sensitivity of the N assay was reassessed in 618 COVID-19 convalescent individuals who were vaccinated at the time of infection. In this vaccinated cohort, a threshold of 99% specificity (34.46 BAU/mL) for N resulted in 63% sensitivity [4]. Lowering the threshold to 90% specificity increased the sensitivity to 88%. Since 97% of participants were vaccinated in this study, to reduce false negatives, the 90% specificity threshold was used to assess seropositivity.

To inform our objective of differentiating between antibodies produced following natural infection and vaccine-induced antibodies, we developed a variable titled ‘suggested status’ based on antigen calls.

*Suggested status:*

- Indeterminate
- No antibody response detected
- Past infection, may also be vaccinated (This referred to a confirmed past infection with/without the vaccine, and is indicated as ‘yes’ in the seroprevalence Table 1)
- Vaccinated or past infection (In this case, we were unable to differentiate between vaccine or past infection, and was indicated as ‘no’ in the seroprevalence Table 1)

[1] Colwill K, Galipeau Y, Stuible M, Gervais C, et al. Clin Transl Immunology. 2022 Mar 23;11(3):e1380. doi: 10.1002/cti2.1380. eCollection 2022.

[2] Murphy TJ, Swail H, Jain J, Anderson M, et al. CMAJ Aug 14, 2023. 195(31) E1030-E1037; DOI: <https://doi.org/10.1503/cmaj.230249>

[3] Breznik JA, Zhang A, Huynh A, Miller MS et al. J Am Med Dir Assoc. 2021 Dec; 22(12):2512-2514. doi: 10.1016/j.jamda.2021.10.001. Epub 2021 Nov 1.

[4] Colwill K, Pasculescu A, Qi F, Abe KT, et al. Determining SArS-CoV-2 infection rates by nucleocapsid seropositivity in a vaccinated population with high seroprevalence. Poster presentation at the COVID-19 Immunity Task Force meeting. 2023. Available at: <https://www.covid19immunitytaskforce.ca/wp-content/uploads/2023/03/A045.pdf>
