# Supplementary material for: Patterns of SARS-CoV-2 seropositivity among essential workers in long term care and retirement homes in Ontario, Canada: A descriptive cross-sectional study
Source: PLOS Glob Public Health. 2025 Mar 28;5(3):e0004294. doi: 10.1371/journal.pgph.0004294 (PMC11952236; doi:10.1371/journal.pgph.0004294)
Supplement: S4 Text — (DOCX) [file pgph.0004294.s004.docx]

**Study Variables**

**Neighbourhood and hotspot variables**

The hotspot variable was created as a dichotomous measure, wherein a hotspot refers to neighbourhoods in Ontario that comprised 20% of the population with the highest per-capita incidence of diagnosed SARS-CoV-2 cases between January 23, 2020 and January 16, 2021. Non-hotspots refer to the remaining geographic areas.

We used the provincial Public Health Case and Contact Management (CCM+) surveillance system of person-level, anonymized surveillance data to obtain dissemination area (DA) characteristics, which are census geographic units representing an average of 400-700 residents. The DA of residence was identified by linking postal codes (obtained via the demographic questionnaire) to publicly available 2016 Canadian Census data. We used this information to generate variables related to DA-level income and proportion of essential workers. Income was defined as the after-tax income per person equivalent, and the definition of the proportion of essential workers who cannot work from home was based on national occupation categories (trades, transport, and equipment operation; sales and services; manufacturing and utilities; resources, agriculture, and production; and health) in each DA[r]. The quintiles or tertiles of these two DA-level socioeconomic factors were ranked within each public health unit (PHU) and adjusted by DA population.

We generated the COVID-19 Hotspots variable using forward sortation area (FSA) information applied in Ontario to prioritize the vaccine strategy. FSA is the first three characters of a postal code. Starting with 526 unique FSAs in Ontario, we merged small population FSAs (less than 1,000) with nearby FSAs to create 509 unique FSAs with neighbourhood risk information in Ontario. We used the cumulative incidence rate of COVID-19 (for the period between January 23, 2020, and January 16, 2021, and excluding cases in long-term and retirement homes) in each FSA as an index to categorize the FSAs into 10 neighbourhood risk groups with equal population.
