# Supplementary material for: Patterns of SARS-CoV-2 seropositivity among essential workers in long term care and retirement homes in Ontario, Canada: A descriptive cross-sectional study
Source: PLOS Glob Public Health. 2025 Mar 28;5(3):e0004294. doi: 10.1371/journal.pgph.0004294 (PMC11952236; doi:10.1371/journal.pgph.0004294)
Supplement: S1 Table — (DOCX) [file pgph.0004294.s005.docx]

***Summary of Data Sources and Variables***

| Data source | Variables collected | Question type | Variable transformation | Final included categories | Prefer not to answer included as an option |
| --- | --- | --- | --- | --- | --- |
| Demographic Questionnaire | Age | open | -- | Age (years) | X |
|  | Gender | MCQ + open | Self-descriptions + prefer not to answer collapsed to protect participant anonymity | Man Woman  Other/prefer not to answer | X |
|  | Education level | MCQ | -- | Up to high school graduation Post secondary Graduate/Post-Graduate | X |
|  | Race | MCQ + open | Data were collapsed into four categories  White-European or North American were collapsed into “White”  Black-African or North American or Afro-Carribbean or European were collapsed into “Black”  SouthEast, South, and East Asian were collapsed into “East or SouthEast Asian”  Latin X, Hispanic, Middle Eastern, West Asian, and multiracial were collapsed into “Other racialized”. | White  Black  East/South-East Asian  Other racialized | X |
|  | Income (household level) | MCQ | Data were collapsed into three categories | $0-$59,000  $60,000-$89,999  $90,000 or greater |  |
|  | Number of people in household | open | Participants were asked “1-How many people currently live in your household?” and “2-What is the total number of unique household members that have lived in your household since the start of the pandemic (March 2020) to now.” The latter was perceived to be a question more aligned with our conceptual framework, however, we had a large percent of missing data (42%). To create this variable, we used the response from Question 2; missing data were supplemented using the response from Question 1. This reduced the missing data to 7%.  Data were collapsed into three categories | 1  2-4  5+ | X |
|  | Housing type | MCQ + open | Data presented as three categories | Apartment/condo  House  Other | X |
|  | Occupation (title) | MCQ + open | Physician, specialist, nurse, and registered practical nurse were collapsed into “Physician/nurse”  PSW, custodial staff, housekeeping staff, kitchen staff, laundry staff were collapsed into “PSW/support staff”  Managers, reception, administrator were collapsed into “Administration/Management”  Allied health professionals (including counselors, behavioural support specialists, IPAC specialists, dieticians, social workers, respiratory therapists, recreational therapists, activities and recreation coordinators, and screeners) were collapsed into “Other” | Physician/nurse  PSW/support staff (e.g., laundry, kitchen)  Administration/management  Other |  |
|  | Employment status | MCQ | Part time workers, agency/contract workers were collapsed | Full-time  Part-time or agency/contract |  |
|  | Transportation to work | MCQ (check all that apply) + open | Data were collapsed into four categories. Because this question asked participants to ‘check all that apply’, the totals in Table 1 exceed 100%.  To assess transmission risk (Table 2), we assigned participants the transportation method that, in our view, would have greater exposure risk (e.g., if participant selected walk to work and use public transport, we assigned public transport as the mode of transportation). | Walk or cycle  Public transport  Rideshare/carpool  Drive alone |  |
|  | Paid sick leave | MCQ | -- | Yes  No  I do not know |  |
| LTCH level questionnaire | Home type | MCQ | -- | LTCH  RH |  |
| Missing/Prefer not to answer |  | | These data were collapsed into ‘missing’ for our analysis. | Missing/unknown |  |

MCQ – multiple choice question
