# Supplementary material for: Patterns of SARS-CoV-2 seropositivity among essential workers in long term care and retirement homes in Ontario, Canada: A descriptive cross-sectional study
Source: PLOS Glob Public Health. 2025 Mar 28;5(3):e0004294. doi: 10.1371/journal.pgph.0004294 (PMC11952236; doi:10.1371/journal.pgph.0004294)
Supplement: S2 Table — Age-adjusted SARS-CoV-2 seroprevalence among LTCH/RH staff. Study figures, R packages used in the methods, code, and figure codes are provided at: https://github.com/mishra-lab/COVID-WellnessHubSeroprevalence.git. (DOCX) [file pgph.0004294.s006.docx]

| ***Age-Adjusted SARS-CoV-2 Seroprevalence among LTCH/RH Staff*** | | | | | |
| --- | --- | --- | --- | --- | --- |
| **Group** | **Label** | **Variable** | **Prevalence ratio** | **2.50%** | **97.50%** |
| Period | Period | Period (baseline: Before 2022-01-01) | Baseline | | |
|  |  | After vs Before 2022-01-01 | 1.87 | 1.44 | 2.43 |
| Socio-demographic and economic characteristics | Gender | Gender (baseline: Man) | Baseline | | |
|  |  | Woman vs Man | 1.48 | 0.97 | 2.25 |
|  | Educational level | Education (baseline: Up to high school graduation) | Baseline | | |
|  |  | College degree or trades certificate vs Up to high school graduation | 1.00 | 0.70 | 1.41 |
|  |  | University bachelor's degree vs Up to high school graduation | 1.15 | 0.83 | 1.61 |
|  |  | Graduate or professional degree vs Up to high school graduation | 0.92 | 0.62 | 1.37 |
|  | Race | Race (baseline: White) | Baseline | | |
|  |  | Black vs White | 1.78 | 1.28 | 2.48 |
|  |  | East or Southeast Asian vs White | 1.55 | 1.18 | 2.04 |
|  |  | Other racialized vs White | 1.42 | 1.03 | 1.96 |
| Household characteristics | Income (household level) | Household income (baseline: $0-$59,999) | Baseline | | |
|  |  | $60,000-$89,999 vs $0-$59,999 | 1.03 | 0.72 | 1.5 |
|  |  | $90,000 or more vs $0-$59,999 | 1.10 | 0.81 | 1.5 |
|  | Number of people in household | Number of people in household (baseline: 1) | Baseline | | |
|  |  | 2-4 vs 1 | 1.10 | 0.70 | 1.73 |
|  |  | 5+ vs 1 | 1.13 | 0.69 | 1.86 |
|  | Housing Type | Housing type (baseline: Apartment/condo) | Baseline | | |
|  |  | House vs Apartment/condo | 0.88 | 0.69 | 1.13 |
|  |  | Other vs Apartment/condo | 0.84 | 0.47 | 1.49 |
| Neighbourhood-level characteristics | FSA-level hotspot/non-hotspot indicator | FSA-level hotspot (baseline: Non-hotspot) | Baseline | | |
|  |  | Hotspot vs Non-hotspot | 1.25 | 1.00 | 1.57 |
|  | DA-level income tertile | DA-level income (baseline: 1-Lowest) | Baseline | | |
|  |  | income 2 vs 1 | 0.89 | 0.68 | 1.15 |
|  |  | income 3 vs 1 | 0.73 | 0.54 | 0.98 |
|  | DA-level essential worker tertile | DA-level proportion essential workers (baseline: 1-Lowest) | Baseline | | |
|  |  | proportion essential workers 2 vs 1 | 1.15 | 0.85 | 1.55 |
|  |  | proportion essential workers 3 vs 1 | 1.23 | 0.91 | 1.65 |
| Occupational characteristics | Occupation (title) | Occupation (baseline: Administration or management) | Baseline | | |
|  |  | Personal support worker or support staff vs Administration or management | 1.17 | 0.87 | 1.58 |
|  |  | Physician, nurse or registered practical nurses vs Administration or management | 1.06 | 0.77 | 1.47 |
|  |  | Other vs Administration or management | 0.89 | 0.59 | 1.33 |
|  | Employment status | Employment status (baseline: Full-time) | Baseline | | |
|  |  | Part-time or agency/contract vs Full-time | 1.00 | 0.77 | 1.28 |
|  |  | Other vs Full-time | 0.92 | 0.52 | 1.65 |
|  | Home Type (LTCH/RH) | Home type (baseline: Long-term care home) | Baseline | Baseline | Baseline |
|  |  | Retirement home vs Long-term care home | 0.90 | 0.69 | 1.19 |
|  | Transportation to work | Transportation to work (baseline: Drive alone) | Baseline | | |
|  |  | Walk or cycle vs Drive alone | 0.80 | 0.44 | 1.47 |
|  |  | Rideshare or carpool vs Drive alone | 0.91 | 0.63 | 1.33 |
|  |  | Public transport vs Drive alone | 1.23 | 0.93 | 1.62 |
|  |  | Work from home, or not currently working vs Drive alone | 1.33 | 0.72 | 2.43 |
|  | Paid Sick Leave | Paid sick leave (baseline: Yes) | Baseline | | |
|  |  | No vs Yes | 0.58 | 0.45 | 0.75 |
