# Supplementary material for: Patterns of SARS-CoV-2 seropositivity among essential workers in long term care and retirement homes in Ontario, Canada: A descriptive cross-sectional study
Source: PLOS Glob Public Health. 2025 Mar 28;5(3):e0004294. doi: 10.1371/journal.pgph.0004294 (PMC11952236; doi:10.1371/journal.pgph.0004294)
Supplement: Attachment — Submitted filename: PGPH-D-24-01292_reviewer-2024.pdf [file pgph.0004294.s008.pdf]

PLOS Global Public Health

Patterns of SARS-CoV-2 seropositivity among essential workers in long term care and retirement homes in Ontario, Canada: A descriptive cross-sectional study

--Manuscript Draft--

**Manuscript Number:**	PGPH-D-24-01292
**Article Type:**	Research Article
**Full Title:**	Patterns of SARS-CoV-2 seropositivity among essential workers in long term care and retirement homes in Ontario, Canada: A descriptive cross-sectional study
**Short Title:**	SARS-CoV-2 seropositivity among LTCH/RH staff
**Corresponding Author:**	Christine Fahim<br>University of Toronto<br>Toronto, CANADA
**Order of Authors:**	Christine Fahim
	Siyi Wang, MSc
	Nimitha Paul, MPH
	Karen Colwill, PhD
	Roya Dayam, PhD
	Jamie M. Boyd, MSc
	Huiting Ma, MSc
	Vincenza Gruppuso, PhD
	Ana Mrazovac, MSc
	Jessica Firman, MPH
	Anjali Patel, BSc
	Vanessa Bach BPH
	Keelia Quinn de Launay, MSc
	Alyson Takaoka, MSc
	Vanja Grubac, MSc
	Anne-Claude Gingras, PhD
	Sharon E. Straus, MD, MSc
	Sharmistha Mishra, MD, MSc, PhD
**Keywords:**	SARS-CoV-2; COVID-19; seroprevalence; dried blood spot; ELISA; Long-Term Care; Retirement Homes; Ontario; Canada
**Abstract:**	Objectives<br><br>Understanding patterns of SARS-CoV-2 seroprevalence among Long-Term Care Home and Retirement Home (LTCH/RH) staff is critical to designing effective public health interventions. We estimated SARS-CoV-2 seroprevalence among LTCH/RH staff in Ontario, Canada between May 2021-October 2022.<br><br>Methods<br><br>We conducted a cross-sectional analysis of SARS-CoV-2 seroprevalence among LTCH/RH staff. Eligible participants completed a demographic questionnaire and provided a dried blood spot sample. Positive seroprevalence was defined as having a SARS-CoV-2 infection and determined using anti-nucleocapsid total IgG antibodies analyzed with a validated chemiluminescent ELISA. We report age-adjusted

Powered by Editorial Manager® and ProduXion Manager® from Aries Systems Corporation

prevalence ratios [PR; confidence interval, CI] by participant socio-demographic, household, neighbourhood, and occupational characteristics and stratified the analyses over two time periods (period 1: 2021-05-17 to 2021-12-31; period 2: 2022-01-02 to 2022-10-25).

Results

A total of 603 staff were included in our analysis; n=235 (39%) were enrolled in period 1 and n=368 (61%) were enrolled in period 2. Seroprevalence was 24% and 44% in periods 1 and 2, respectively. Age-adjusted prevalence ratios were nearly 2-fold higher among Black [PR 1.78; CI 1.28-2.48] East and Southeast Asian [PR 1.55, CI 1.18-2.04] and other racialized participants [PR 1.42, CI 1.03-1.96] compared to White participants. We did not observe a pattern across household characteristics, although we observed a trend towards higher seropositivity among participants living in COVID-19 hotspots. Prevalence ratios were lower for participants in higher income neighbourhoods [PR 0.72, CI 0.58-0.98]. We did not observe variability in seroprevalence across occupational characteristics with the exception of paid sick leave which was higher among participants with home-provided paid sick leave at the time of the survey [PR 0.58, CI 0.45-0.75].

Discussion

Among LTCH/RH staff, we found important sources of variability of SARS-CoV-2 seroprevalence and strong correlations with socioeconomic disparities. Our findings show the importance of designing equity-rooted health interventions that recognize the intersection between community and the workplace.

Additional Information:	
**Question**	**Response**
**Financial Disclosure**<br><br>Enter a financial disclosure statement that describes the sources of funding for the work included in this submission and the role the funder(s) played. This includes grants and any commercial funding of the work or authors.<br><br>This statement will be typeset if the manuscript is accepted for publication.<br><br>*Please review the submission guidelines and the instructions link below for detailed requirements and guidance.*	This study was funded by the COVID-19 Immunity Task Force via the Public Health Agency of Canada (#2021-HQ-000143). SM (CRC number 950-232643) is supported by a Canada Research Chair (Tier 2) in Mathematical Modeling and Program Science. SES holds a Canada Research Chair (Tier 1) in in Knowledge Translation and Quality of Care. ACG holds a Canada Research Chair (Tier 1) in Functional Proteomics.
**Competing Interests**<br><br>On behalf of all authors, disclose any competing interests that could be perceived to bias this work.	None.

Powered by Editorial Manager® and ProduXion Manager® from Aries Systems Corporation

This statement will be typeset if the manuscript is accepted for publication.<br><br>*Please review the instructions link below and PLOS Global Public Health's competing interests policy to determine what information must be disclosed at submission.*	
**Data Availability**<br><br>Before publication, Authors are required to make fully available and without restriction all data underlying their findings. Please see our PLOS Data Policy page for detailed information on this policy.<br><br>A **Data Availability Statement**, detailing where the data can be accessed, is required at first submission. Insert your Data Availability Statement in the box below.<br><br>Please see the data reporting section of our submission guidelines for instructions on what you need to include in your Data Availability Statement.<br><br>This statement will be typeset if the manuscript is accepted for publication.<br><br>*PLOS allows rare exemptions to address legal and ethical concerns. If you have legal or ethical restrictions, please use the box below to detail these in full sentences for the Journal team to consider.*	Our datasets were provided to the COVID-19 Immunity Task Force CITF Databank. Access to the data can be requested via: https://portal.citf.mcgill.ca/

Powered by Editorial Manager® and ProduXion Manager® from Aries Systems Corporation

Manuscript
Click here to access/download;Manuscript;Serostatus Paper_full_for submission_20240531_Clean.docx

Full title: Patterns of SARS-CoV-2 seropositivity among essential workers in long term care and retirement homes in Ontario, Canada: A descriptive cross-sectional study

Short title: SARS-CoV-2 seropositivity among LTCH/RH staff

Christine Fahim[1,2], PhD MSc*, Siyi Wang MSc[1], Nimitha Paul MPH[1], Karen Colwill[3], PhD, Roya Dayam[3], PhD, Jamie M. Boyd MSc[1], Huiting Ma MSc[1], Vincenza Gruppuso PhD[1], Ana Mrazovac MSc[1], Jessica Firman[1] MPH, Anjali Patel BSc[1], Vanessa Bach BPH[1], Keelia Quinn de Launay MSc[1], Alyson Takaoka MSc[1], Vanja Grubac MSc[1], Anne-Claude Gingras[2,3], PhD, Sharon E. Straus MD MSc[1,2,5], Sharmistha Mishra MD, MSc, PhD[1,4-7]

1 – Li Ka Shing Knowledge Institute, Unity Health Toronto, Toronto, Canada
2 – Dalla Lana School of Public Health, University of Toronto
3 – Lunenfeld-Tanenbaum Research Institute, Sinai Health, Toronto, Canada
4 – Department of Molecular Genetics, University of Toronto, Toronto, Canada
5 – Department of Medicine, University of Toronto
6 – Institute of Medical Sciences, University of Toronto
7 – Institute of Clinical and Evaluative Sciences, Toronto

*Corresponding author:

Christine Fahim, PhD, MSc
Rm. 722, Li Ka Shing Knowledge Institute, St. Michael’s Hospital, Unity Health Toronto
30 Bond Street, Toronto, Ontario, Canada, M5B 1W8
Tel: 1-416-360-4000 x77300
Email: christine.fahim@unityhealth.to

Word Count: [3,076]
Tables: [1]
Figures: [1]
Appendix: [6]

Abstract

Objectives: Understanding patterns of SARS-CoV-2 seroprevalence among Long-Term Care Home and Retirement Home (LTCH/RH) staff is critical to designing effective public health interventions. We estimated SARS-CoV-2 seroprevalence among LTCH/RH staff in Ontario, Canada between May 2021-October 2022.

Methods: We conducted a cross-sectional analysis of SARS-CoV-2 seroprevalence among LTCH/RH staff. Eligible participants completed a demographic questionnaire and provided a dried blood spot sample. Positive seroprevalence was defined as having a SARS-CoV-2 infection and determined using anti-nucleocapsid total IgG antibodies analyzed with a validated chemiluminescent ELISA. We report age-adjusted prevalence ratios [PR; confidence interval, CI] by participant socio-demographic, household, neighbourhood, and occupational characteristics and stratified the analyses over two time periods (period 1: 2021-05-17 to 2021-12-31; period 2: 2022-01-02 to 2022-10-25).

Results: A total of 603 staff were included in our analysis; n=235 (39%) were enrolled in period 1 and n=368 (61%) were enrolled in period 2. Seroprevalence was 24% and 44% in periods 1 and 2, respectively. Age-adjusted prevalence ratios were nearly 2-fold higher among Black [PR 1.78; CI 1.28-2.48] East and Southeast Asian [PR 1.55, CI 1.18-2.04] and other racialized participants [PR 1.42, CI 1.03-1.96] compared to White participants. We did not observe a pattern across household characteristics, although we observed a trend towards higher seropositivity among participants living in COVID-19 hotspots. Prevalence ratios were lower for participants in higher income neighbourhoods [PR 0.72, CI 0.58-0.98]. We did not observe variability in seroprevalence across occupational characteristics with the exception of paid sick leave which was higher among participants with home-provided paid sick leave at the time of the survey [PR 0.58, CI 0.45-0.75].

Discussion: Among LTCH/RH staff, we found important sources of variability of SARS-CoV-2 seroprevalence and strong correlations with socioeconomic disparities. Our findings show the importance of designing equity-rooted health interventions that recognize the intersection between community and the workplace.

Keywords: SARS-CoV-2, COVID-19, seroprevalence, dried blood spot, ELISA, Long-Term Care, Retirement Homes, Ontario, Canada

Introduction

Staff working in LTCH/RH represent a diversity of occupational and socio-demographic characteristics and experiences which could shape exposure risks as they relate to SARS-CoV-2 acquisition. Occupations in LTCH/RH range from regulated and higher-wage positions (e.g., physicians, nurse practitioners, and management) to unregulated and lower-wage positions such as personal support workers or care aides(1). Unregulated workers do not have mandatory regulations, training, or registration, often resulted in decreased job protections, benefits and pay(2,3). It is estimated that personal support workers and care aides provide 90% of the direct, person-to-person, care for residents in LTCH/RH(3–5).

In Ontario, Canada, nearly half of all personal support workers are employed in LTCH(6). Recent data show that approximately 90% of personal support workers in the greater Toronto Area identify as racially minoritized, and 55% report living in lower income households (6). Less is known about the remainder of the workforce in LTCH/RH. These above studies have made important contributions to understanding the experiences of subsets of LTCH staff, but none to date have characterized occupational and household characteristics across the range of the LTCH and RH workforce.

An extensive body of work on the SARS-CoV-2 epidemic in Canada has documented the complex systemic and multisectoral factors that shaped social determinants of health, including occupational health, and household characteristics(7–9). These factors have been shown to drive social and physical networks and workplace safety and security (e.g. transportation to/from onsite work, infection control resources, services, and institutional policies and programmes), which in turn, collectively shape SARS-CoV-2 acquisition and transmission risks among essential workers. Yet, to date, there has not been a description of the pattern of SARS-CoV-2

risks, measured using seroprevalence, among LTCH and RH staff in Canada, particularly after the early waves of the epidemic in 2020 and after vaccine(10) roll-out.

We therefore sought to characterise patterns of seroprevalence among LTCH/RH workers in Ontario between May 2021 and October 2022, after vaccine roll-out; and across socio-demographic, household, neighbourhood and occupational characteristics.

Methods

Study design and period

We conducted an observational, cross-sectional study using baseline data from the Wellness Hub study. The Wellness Hub study is a prospective cohort study of Ontario, Canada LTCH/RH staff, residents, and caregivers recruited and enrolled into the cohort between May 17, 2021 and October 25, 2022. The Wellness Hub study was designed to measure seroprevalence of SARS-CoV-2 antibodies among LTCH and RH staff, residents, and caregivers, with recruitment from LTCH and RH. In this paper, we use the baseline data from LTCH/RH staff cohort, and restricted to participants residing in the Greater Toronto Area and its surrounding areas (e.g., Hamilton). We report our study findings using the Strengthening the Reporting of Observational Studies in Epidemiology checklist.

Sampling frame and sampling strategy

Wellness Hub study sites were recruited using both purposive and convenience sampling. Recruitment strategies included both active (e.g., tailored presentations to LTCH/RH networks) and passive (e.g., website/newsletter advertisements, word of mouth) strategies. We leveraged an extensive network of project partners to disseminate the study materials and facilitate recruitment (Appendix 1). Within recruited homes, we recruited staff using convenience and snowball

sampling. Recruitment strategies included: emails and phone calls to LTCH/RH managers and staff, advertisements via posters, newsletters, on-site recruitment days, social media, and study self-enrollment via website URLs and QR codes.

Setting

We recruited 72 LTCH/RH to participate in the Wellness Hub study. Homes were eligible to participate in Wellness Hub if they agreed to participate in individual-level recruitment, a home-level needs assessment interview to address challenges facing their home during the COVID-19 pandemic and if they identified a designated ‘point person’ to act as a primary contact for the study. Indigenous LTCH/RH and Indigenous participants were excluded from the study, given resource and experience gaps on our study team to appropriately engage members of Indigenous communities. Within all participating homes, four LTCH/RH populations (staff, household members of staff, residents, and essential care partners of residents [e.g. family, caregivers]) were eligible to participate in the study by providing DBS samples and completing an in-depth questionnaire; in this article, we present the findings from the LTCH/RH staff cohort.

Participants

Participants were eligible for inclusion if they: identified as a staff (including full-time, part-time paid staff and unpaid volunteers) working at a LTCH/RH at the time of consent and enrollment; were comfortable speaking/reading English or French; were at least 18 years old; were willing to complete a study demographic questionnaire and provide a DBS sample. Participants who self-identified as Indigenous in the study consent or data collection form were not eligible to participate.

Data Collection

Participants completed a demographic questionnaire (Appendix 2) and provided a DBS sample. Demographic questionnaires included multiple choice and open-ended questions, and were completed using one of the following methods: 1) a hard-copy printed version of the questionnaire, 2) an online version of the questionnaire via REDCap platform or 3) a questionnaire through an in-person and/or virtual call led by a research team member. Participants provided DBS samples via one of the following methods: 1) collected in-person with the support of study staff or LTCH/RH staff, or 2) self-collected by participants following detailed instructions. Participants had the option of leaving their self-collected sample at a drop-off site within the study home or mailing the sample back to the study team.

DBS Sample Storage and Testing

DBS were collected on Whatman 903 cards using a blade lancet for finger pricking. No identifying information was listed on the cards, except a unique participant ID. Collection of ≥2 filled spots on the card was requested. Cards were then air dried (according to the manufacturer's instructions) and placed in individual sealable bags, along with a desiccant pouch. Cards were transferred to the Gingras laboratory at Sinai Health. Upon reception in that lab, the cards were placed at 4°C for short-term storage, or frozen (at -80°C) for long-term storage. Cards were then brought to room temperature before the packages were opened, and a portion of the spot (2 x 3 mm or a 1 x 3 mm diameter circle if the sample was limited as this was sufficient for one pass at the assays if the blood soaked through the Whatman paper) was then extracted with a semi-automated hole puncher directly into a 96-well plate.

Outcome measure

Our primary outcome was seropositivity determined using DBS, based on anti-nucleocapsid total IgG antibodies by a validated chemiluminescent ELISA. In alignment with another

seroprevalence study conducted in LTCH during this time period(11), we performed our analyses at a specificity threshold of 90% (a relative ratio to a synthetic standard of 0.341; 11.34 binding antibody units [BAU/mL], corresponding to 88% sensitivity in a vaccinated cohort, which provides the best predictive value (negative predictive value * positive predictive value) when seroprevalence is 40% or greater(12). As noted in several studies, many individuals who have breakthrough infection after vaccination either do not seroconvert for nucleocapsid or had lower antibody levels to it(13). Thus, we used the relative ratio nucleocapsid threshold of 0.341 (BAU of 11.34 BAU/mL) to reduce false negatives. DBS passing the threshold of seropositivity for anti-nucleocapsid IgG antibodies were identified as having a natural COVID-19 infection and were marked as 'seropositive for natural infection'; DBS that did not pass the threshold were considered 'seronegative for natural infection' (see Appendix 3 for more detail).

Conceptual framework and variables

Our conceptual framework in selecting variables to examine patterns of seroprevalence was informed by literature on epidemiological and socioeconomic factors associated heightened risks of SARS-CoV-2 acquisition in Canada; and literature more specific to exposure risks in occupational settings(9,14–16). We classified variables of interest according to four domains conceptualized based on potential contexts that shape exposure risks for SARS-CoV-2 among LTCH/RH staff: sociodemographic, household characteristics, neighbourhood characteristics, and occupational characteristics. Sociodemographic characteristics included: age(17–19), gender(20), educational attainment(21); and race as a proxy for individual, institutional, and structural racism(9,21–23). Household characteristics included: household income(8), household density(9,21), and housing type(21,24,25) (Appendix 4). Neighbourhood-level characteristics refer to the residential neighbourhood of the participant (not the home) and include:

socioeconomic indicators of neighbourhood-level median household income (measured as tertile) and proportion essential workers (measured as tertile). The tertiles for neighbourhood-level median household income and proportion essential workers were based on data at the level of the dissemination area from the 2016 Census from Statistics Canada(26), with details included in Appendix 5. We also included a neighborhood-level measure of community transmission, defined as "hotspot versus non-hotspot" using the neighbourhood-level cumulative SARS-CoV-2 diagnoses. We used the first three digits of participant postal code of residence (called the forward sortation area) to create the neighbourhood-level hotspot variable as a dichotomous measure. A hotspot referred to neighbourhoods (defined using the forward sortation area) in Ontario that comprised 20% of the population with the highest per-capita incidence of diagnosed SARS-CoV-2 cases between January 23, 2020, and January 16, 2021(27,28) . We defined the remaining geographical areas as non-hotspots. Appendix 5 details the derivation of neighbourhood-level variables including hotspots, dissemination area characteristics, and forward sortation area levels.

Finally, our last domain centered on occupational-related characteristics, and included: occupational role, employment status, type of home (LTCH or RH), transportation used to attend the home for work, and current paid sick leave. We classified occupational role using the self-reported answer to "what best describes your job title at this LTCH/RH" and regrouped open text responses based on best fit to the categories. We classified employment status using the self-reported answer to: "what is your employment status in this LTCH/RH" with open text responses coded into existing categories. For transportation, we grouped modes of transportation to the home based on anticipated magnitude of contacts during occupation-related movement from/to households from workplaces: few contacts (i.e., working from household or remote work) to

greater number of contacts (i.e., use of rideshares or carpooling; use of public transportation). The variable of current paid sick leave at the home of employment was generated using self-reported answer to: “do you have paid sick leave at this facility”. If individuals answered “no”, “do not know / unsure”, or if responses were missing, we categorized their response as “no” for current paid sick leave.

Analysis plan

Survey data with responses fewer than n=5 were collapsed in order to protect participant anonymity (Appendix 2). Open-ended responses were independently themed by a Research Scientist (CF) and Research Coordinator (NP) to facilitate analysis. Continuous data were reported using means, standard deviations (SD) and medians, and inter-quartile range (IQR). We conducted descriptive analyses and reported seropositivity (i.e. seroprevalence) by the covariates of interest. We then generated age-adjusted prevalence ratios (PR) with 95% confidence intervals (95% CI) for each covariate using normal approximation (Wald), and using *a priori* selection of the reference category as appropriate. For age-adjustment, we applied logistic regression with the log link function for the PR, where the outcome was seroprevalence and included the age as a continuous variable. Data cleaning, analyses, and visualization were conducted in R 4.3.0.

Research Ethics

This study was approved by the Unity Health Toronto Research Ethics Board (REB 20-347). Participants provided formal consent between May 6 2021 and June 26 2023. All participants or their shared decision maker provided either written (hard copy) or online (captured via REDCap) consent. There were no minors involved in the study (participant age range 18-79 years).

Results

Participant characteristics

Of the n=797 LTCH/RH staff who participated in the Wellness Hub study and completed a demographic questionnaire, n=603 provided a DBS and were included in this analysis. Among the 603 participants, 235 (39%) were enrolled in period 1 and 368 (61%) in period 2. Nearly all (97.7%) participants reported receipt of at least 2 doses or equivalent of SARS-CoV-2 vaccination (37.1% of participants in period 1 and 60.8% in period 2). Table 1 summarizes participant characteristics under the conceptual framework. Eighty-three percent of the participants identified as women, 12% as men, and 4% chose not to specify. Nearly half (44%) of participants identified as White, 24% as East or Southeast Asian, 10% as Black, and 16% identified as a member of a non-Black, non-East/Southeast Asian racialized community (Table 1). The crude SARS-CoV-2 seroprevalence in our sample was 24% in period 1 and 44% in period 2 (Table 1, Appendix 6).

Age-adjusted pattern of seroprevalence

Seroprevalence was nearly 2-fold higher in period 2 compared to period 1 [PR 1.87; CI 1.44-2.43]. Seropositivity was nearly 2-fold higher among racialized participants compared with White participants: the prevalence ratio for seropositivity among Black participants was 1.78 [CI 1.28-2.48], 1.55 [CI 1.18-2.04] among East/Southeast Asian participants, and 1.42 [CI 1.03-1.96] among other racialized participants. Although there were few men in our study, there was a trend towards a 1.5-fold higher seroprevalence among women [PR 1.48, CI 0.97-2.25] and similar seroprevalence by levels educational attainment (Appendix 7).

We did not observe a pattern across household characteristics. There was a meaningful trend towards higher seropositivity among participants residing in hotspots compared with non-hotspots [PR 1.25, CI 1.00-1.57]. Prevalence ratios were lower for participants living in higher income neighbourhoods [PR 0.72, CI 0.58 – 0.98].

We did not observe variability in seroprevalence across occupational characteristics, with the exception of paid sick leave. Seropositvity was higher among participants with paid sick via the home at the time of the survey [PR 0.58, CI 0.45-0.75].

Discussion

Baseline data from a cohort study of LTCH and RH staff in Toronto, Canada demonstrated that SARS-CoV-2 seroprevalence was 24% between May-December 2021 and 44% between January-October 2022. We documented racial disparities, with age-adjusted prevalence nearly two-fold higher among Black and East/Southeast Asian staff as compared with White staff. Seroprevalence was also higher among staff living in COVID-19 hotspots. We did not observe variability by household or occupational factors, except for paid sick leave: staff with higher seroprevalence were more likely to report access to paid sick leave.

The patterns of seroprevalence among LTCH/RH staff are reflective of underlying disparities that have been extensively documented in Canada. Racially minoritized communities experienced some of the highest rates of SARS-CoV-2 infections due to disproportionate exposure risks, including through occupational risks. In addition to the burnout, fatigue, and stress often experienced by LTCH/RH staff, racialized staff further contend with racism and discrimination in the workplace. A 2009 Ontario survey with PSWs showed that over half of PSWs experience harassment or workplace violence; 15% of PSWs experienced violence and harassment related to their(4) race; other studies have provided much greater estimates of racism and workplace harassment suggesting these experiences are likely underreported(29). Within long-term care, racialized workers are overrepresented as frontline caregivers and hold fewer management and administrative roles. The nature of unregulated, precarious work coupled with power imbalances and occupational hierarchies provide limited protections or options(29) for staff, particularly those experiencing racism or discrimination(30) in the workplace.

Seroprevalence was higher among staff with paid sick leave. Although studies are not yet available in Canada, the provision of emergency sick leave in the United States was associated

with reduced SARS-CoV-2 transmission at a community-level(31). Access to sick leave has also been shown to reduce presenteeism with other respiratory illnesses(31). Our finding of higher seroprevalence among staff who had paid sick leave provided by the LTCH, may reflect the fact that sick-leave represents high risks of SARS-CoV-2 exposures, and we did not examine confounders in our descriptive study of patterns. Furthermore, individuals with sick leave may not necessarily be in a position (financially, or other reasons including power hierarchies in occupations such as health care(29)) to take sick leave when needed. Of note however, paid sick-leave is mostly relevant for onward transmission in the LTCH, not for acquisition at the individual-level; and our measures of seroprevalence reflect acquisition. Our study was not designed to examine whether sick-leave was associated with lower level of transmission; if most acquisition among staff occur in the household then sick-leave would not necessarily be associated with seroprevalence. A better measure would be to examine association between seropositivity and the proportion of staff in the LTCH who have paid sick-leave, but this measure was not available in our study.

The neighborhood-level correlation between staff seroprevalence and SARS-CoV-2 hotspots signal the importance of community-level factors and community-level transmission when considering exposure risks among LTCH/RH workers. An earlier study in Ontario found that the spatial distribution of SARS-CoV-2 cases among LTCH workers were very closely aligned with the overall community-level distribution of cases(32). Studies from the United States also suggest that LTCH workers may have been more likely to live in neighborhoods with higher rates of SARS-CoV-2(33). Taken together, the implication of these findings is that reducing community-level transmission is an important component of reducing risks among LTCH/RH staff and outbreaks in LTCH/RH. For example, community-level vaccination programs and other

efforts to reduce community-level transmission (testing, isolation support) prioritized and tailored to SARS-CoV-2 geographical hotspots simultaneously with prioritization of workers within LTCH/RH could potentially be more effective than focusing on homes in isolation of disparities that connect community-transmission and workplace exposure risks. Another implication is that community transmission risks need to be accounted for when evaluating the potential nosocomial transmission impact of policies instituted at the LTCH/RH level. Thus, our findings confirm previous findings and support the need for policies, programs, and pandemic preparedness and response frameworks that are designed to address unmet prevention needs at the intersections between community and the workplace(32,33). Although focused on SARS-CoV-2, the policy implications are also relevant for seasonal influenza and other respiratory virus outbreaks and epidemics(34).

Limitations

Our study is limited to staff who worked in homes that participated in the Wellness Hub study, and thus geographic scope was restricted to the Greater Toronto Area (a largely urban setting, with a larger proportion of racialized population, and where SARS-CoV-2 transmission during the study period was also higher than in more rural settings). As such, our findings may not be generalizable to less urban settings. We were also limited by grouping our examination of associations across wide ranges of time because participants were recruited over 17 months. Another limitation is measurement bias in our outcome of interest, as we did not have data on polymerase-chain-reaction SARS-CoV-2 testing data to confirm and validate the results from the DBS samples. We may have underestimated past infections because some vaccinated individuals may not have produced sufficient anti-nucleocapsid antibodies to pass the positivity threshold or their antibody levels may have waned below the level of detection at the timing of testing.

Finally, the results of our descriptive analyses should be interpreted as observations around variability in patterns of seroprevalence; future study is needed to conduct risk factor and potentially causal analyses.

Conclusions

In conclusion, there exist important sources of variability in SARS-CoV-2 seroprevalence among LTCH/RH staff in Ontario. Findings underscore the importance of developing equity-rooted interventions that address the intersection between community and workplace exposure risks for SARS-CoV-2 and other respiratory viruses.

Acknowledgements: We acknowledge Melanie Delgado-Brand, Tulunay Tursun, Geneviève Mailhot, Martina Tersigni, Adrian Pasculescu, and Freda Qi of the Gingras laboratory for their role in ELISA analysis. Anne-Claude Gingras leads the Functional Genomics and Structure-Function Pillar of CoVaRR-Net. Antigens, protein standards and secondary antibodies for ELISA were kindly provided by The Pandemic Response Challenge Program of the National Research Council of Canada (Dr. Yves Durocher); positive and negative control samples for ELISA assay calibration were from the National Microbiology Laboratory, Public Health Agency of Canada (Dr. John Kim). The robotics equipment used is housed in the Network Biology Collaborative Centre at the Lunenfeld-Tanenbaum Research Institute, a facility supported by the Canada Foundation for Innovation, the Ontario Government, and Genome Canada and Ontario Genomics (OGI-139). We thank Linwei Wang (Unity Health Toronto) for R scripts that were adapted to generate Figure 1. Finally, we would like to thank all of the organizational partners of the Wellness Hub program for their support of this study. A full list of partner organizations can be found at https://wellness-hub.ca/. The authors thank Andreea Manea for supporting manuscript preparation.

Conflicts of Interest: None.

Funding: This study was funded by the COVID-19 Immunity Task Force via the Public Health Agency of Canada (#2021-HQ-000143). SM (CRC number 950-232643) is supported by a Canada Research Chair (Tier 2) in Mathematical Modeling and Program Science. SES holds a Canada Research Chair (Tier 1) in in Knowledge Translation and Quality of Care. ACG holds a Canada Research Chair (Tier 1) in Functional Proteomics.

Supporting Information: Supplementary Files 1 (Appendices 1, 3-6) and 2 (Appendix 2)

Group	Label	Variable	Negative - N=386 (column proportion)	Positive - N=217 (column proportion)	Total - N=603 (column proportion)	Seroprevalence (Positive/Total)
Period	Time period (before/after 2022-01-01)	First period	179 (46%)	56 (26%)	235 (39%)	24%
		Second period	207 (54%)	161 (74%)	368 (61%)	44%
Socio-demographic characteristics	Age	Min / Max	18.0 / 97.0	18.0 / 74.0	18.0 / 97.0	
		Med [IQR]	47.0 [34.0;56.0]	47.0 [36.0;55.0]	47.0 [35.0;56.0]	
		Mean (std)	45.5 (13.8)	45.6 (12.4)	45.5 (13.3)	
		N (exclude missing/unknown)	371	209	580	36%
		Missing/Unknown	15	8	23	35%
	Age group	18-24	32 (8%)	9 (4%)	41 (7%)	22%
		25-34	62 (16%)	39 (18%)	101 (17%)	39%
		35-44	63 (16%)	43 (20%)	106 (18%)	41%
		45-54	106 (27%)	62 (29%)	168 (28%)	37%
		55-65	89 (23%)	47 (22%)	136 (23%)	35%
		65+	19 (5%)	9 (4%)	28 (5%)	32%
		Missing/unknown	15 (4%)	8 (4%)	23 (4%)	35%
	Gender	Man	57 (15%)	18 (8%)	75 (12%)	24%
		Woman	315 (82%)	186 (86%)	501 (83%)	37%
		Missing/unknown	14 (4%)	13 (6%)	27 (4%)	48%
	Educational level	Up to high school graduation	66 (17%)	34 (16%)	100 (17%)	34%
		College degree or trades certificate	118 (31%)	62 (29%)	180 (30%)	34%
		University bachelor's degree	113 (29%)	74 (34%)	187 (31%)	40%
		Graduate or professional degree	73 (19%)	34 (16%)	107 (18%)	32%
		Missing/unknown	16 (4%)	13 (6%)	29 (5%)	45%
	Race	White	190 (49%)	77 (35%)	267 (44%)	29%

		Black	31 (8%)	30 (14%)	61 (10%)	49%
		East or Southeast Asian	84 (22%)	63 (29%)	147 (24%)	43%
		Other racialized	60 (16%)	37 (17%)	97 (16%)	38%
		Missing/unknown	21 (5%)	10 (5%)	31 (5%)	32%
Household characteristics	Income (household level)	$0 - $59,999	77 (20%)	42 (19%)	119 (20%)	35%
		$60,000 - $89,999	60 (16%)	35 (16%)	95 (16%)	37%
		$90,000 or more	121 (31%)	77 (35%)	198 (33%)	39%
		Missing/unknown	128 (33%)	63 (29%)	191 (32%)	33%
	Number of people in household	1	30 (8%)	14 (6%)	44 (7%)	32%
		2-4	261 (68%)	146 (67%)	407 (67%)	36%
		5+	73 (19%)	42 (19%)	115 (19%)	37%
		Missing/unknown	22 (6%)	15 (7%)	37 (6%)	41%
	Housing Type	Apartment/condo	86 (22%)	57 (26%)	143 (24%)	40%
		House	253 (66%)	136 (63%)	389 (65%)	35%
		Other	18 (5%)	9 (4%)	27 (4%)	33%
		Missing/unknown	29 (8%)	15 (7%)	44 (7%)	34%
Neighbourhood-level characteristics	FSA-level hotspot/non-hotspot indicator	Non-hotspot	247 (64%)	121 (56%)	368 (61%)	33%
		Hotspot	115 (30%)	81 (37%)	196 (33%)	41%
		Missing/unknown	24 (6%)	15 (7%)	39 (6%)	38%
	DA-level income tertile	1	116 (30%)	79 (36%)	195 (32%)	41%
		2	123 (32%)	68 (31%)	191 (32%)	36%
		3	118 (31%)	49 (23%)	167 (28%)	29%
		Missing/unknown	29 (8%)	21 (10%)	50 (8%)	42%
	DA-level essential worker tertile	1	107 (28%)	48 (22%)	155 (26%)	31%
		2	132 (34%)	73 (34%)	205 (34%)	36%
		3	118 (31%)	75 (35%)	193 (32%)	39%
		Missing/unknown	29 (8%)	21 (10%)	50 (8%)	42%
Occupational characteristics	Occupation (title)	Administration or management	83 (22%)	44 (20%)	127 (21%)	35%

		Personal support worker or support staff (e.g. kitchen, housekeeping, laundry)	115 (30%)	74 (34%)	189 (31%)	39%
		Physician, nurse or registered practical nurses	91 (24%)	55 (25%)	146 (24%)	38%
		Other	62 (16%)	27 (12%)	89 (15%)	30%
		Missing/unknown	35 (9%)	17 (8%)	52 (9%)	33%
	Employment status	Full-time	252 (65%)	141 (65%)	393 (65%)	36%
		Part-time or agency/contract	99 (26%)	54 (25%)	153 (25%)	35%
		Other	16 (4%)	8 (4%)	24 (4%)	33%
		Missing/unknown	19 (5%)	14 (6%)	33 (5%)	42%
	Home Type (LTCH/RH)	Long-term care home	298 (77%)	173 (80%)	471 (78%)	37%
		Retirement home	88 (23%)	44 (20%)	132 (22%)	33%
	Transportation to work	Drive alone	235 (61%)	127 (59%)	362 (60%)	35%
		Walk or cycle	21 (5%)	8 (4%)	29 (5%)	28%
		Rideshare or carpool (including taxi or uber)	49 (13%)	22 (10%)	71 (12%)	31%
		Public transport	58 (15%)	41 (19%)	99 (16%)	41%
		Work from home, or not currently working	7 (2%)	6 (3%)	13 (2%)	46%
		Missing/unknown	16 (4%)	13 (6%)	29 (5%)	45%
	Paid Sick Leave	Yes	143 (37%)	120 (55%)	263 (44%)	46%
		No	185 (48%)	64 (29%)	249 (41%)	26%
		Missing/unknown	58 (15%)	33 (15%)	91 (15%)	36%

Table 1: Demographic Characteristics

Figure 1: Age-adjusted prevalence ratios

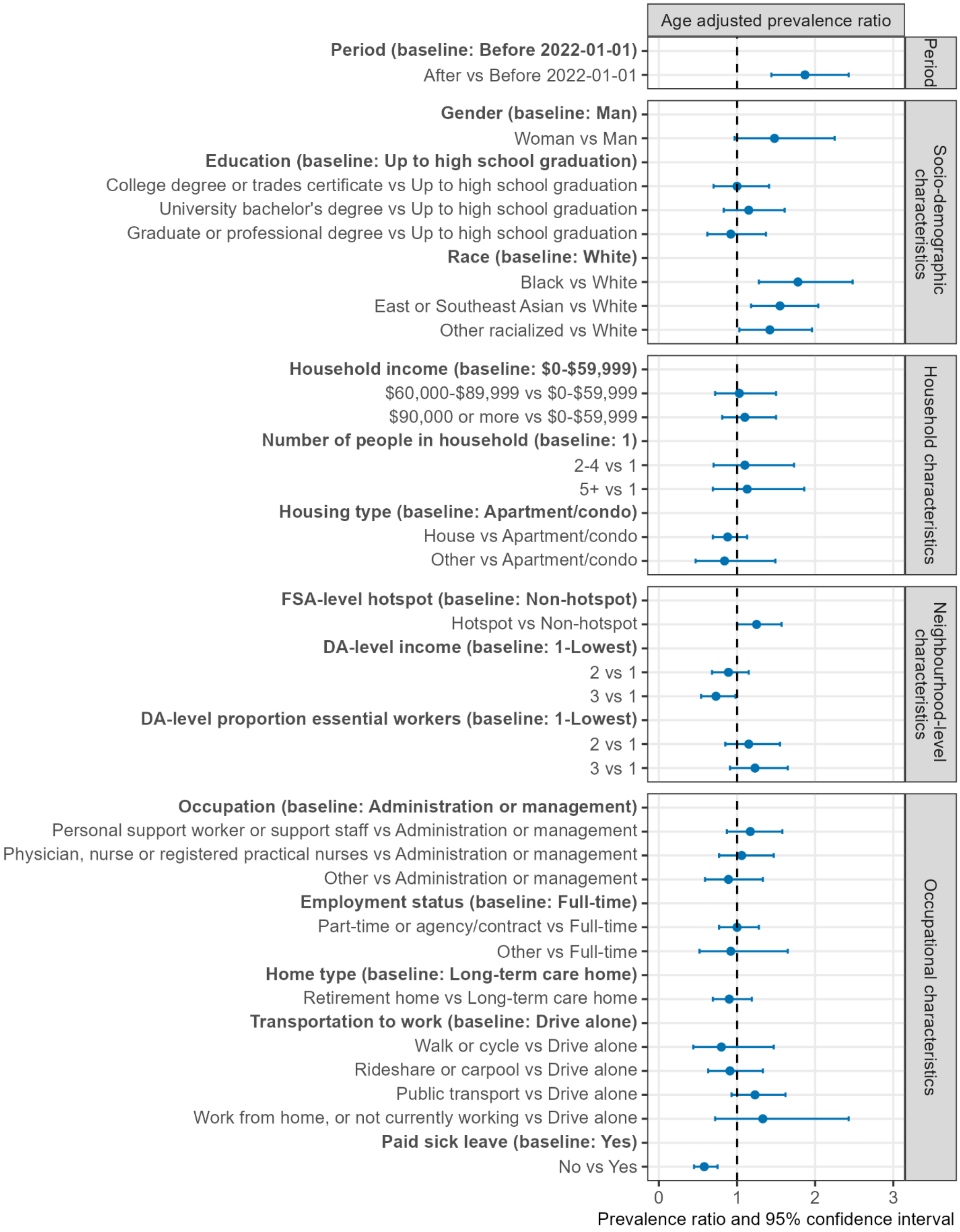

REFERENCES

1. Government of Ontario. Regulations Pertaining to the Health and Supportive Care Providers Oversight Authority (the Authority) [Internet]. Dec 1, 2023. Available from: https://www.ontariocanada.com/registry/view.do?postingId=45993&language=en

2. Ontario Hospital Association. Backgrounder Proposed Regulatory Framework for Personal Support Workers and Related Regulatory Proposal Under the Fixing Long-Term Care Act, 2021 [Internet]. 2023 [cited 2024 May 30]. Available from: https://www.oha.com/Legislative%20and%20Legal%20Issues%20Documents1/PSW%20Regulatory%20Framework%20LTC%20Proposals.pdf

3. Long-Term Care Staffing Study Advisory Group. Long-Term Care Staffing Study [Internet]. Ministry of Long-Term Care; 2020 [cited 2024 May 30]. Available from: https://www.ontario.ca/page/long-term-care-staffing-study

4. Lum J, Sladek J, Ying A, Holloway Payne L. Ontario Personal Support Workers in Home and Community Care: CRNCC/PSNO Survey Results [Internet]. 2010 [cited 2024 May 30]. Available from: https://www.torontomu.ca/content/dam/crncc/knowledge/infocus/factsheets/InFocus-Ontario%20PSWs%20in%20Home%20and%20Community%20Care.pdf

5. Estabrooks CA, Straus SE, Flood CM, Keefe J, Armstrong P, Donner GJ, et al. Restoring trust: COVID-19 and the future of long-term care in Canada. Blais JM, editor. FACETS [Internet]. 2020 Jan 1 [cited 2024 May 30];5(1):651–91. Available from: http://www.facetsjournal.com/doi/10.1139/facets-2020-0056

6. Pinto AD, Hapsari AP, Ho J, Meaney C, Avery L, Hassen N, et al. Precarious work among personal support workers in the Greater Toronto Area: a respondent-driven sampling study. CMAJ Open [Internet]. 2022 Apr [cited 2024 May 30];10(2):E527–38. Available from: http://cmajopen.ca/lookup/doi/10.9778/cmajo.20210338

7. Ontario Agency for Health Protection and Promotion (Public health Ontario). COVID-19 – What We Know So Far About… Social Determinants of Health [Internet]. 2020 [cited 2024 May 30]. Available from: https://www.publichealthontario.ca/-/media/documents/ncov/covid-wwksf/2020/05/what-we-know-social-determinants-health.pdf?la=en#:~:text=Social%20determinants%20of%20health%20(SDOH,ability%20to%20maintain%20physical%20distancing

8. Mishra S, Ma H, Moloney G, Yiu KCY, Darvin D, Landsman D, et al. Increasing concentration of COVID-19 by socioeconomic determinants and geography in Toronto, Canada: an observational study. Ann Epidemiol [Internet]. 2022 Jan [cited 2024 May 30];65:84–92. Available from: https://linkinghub.elsevier.com/retrieve/pii/S1047279721002167

9. Sundaram ME, Calzavara A, Mishra S, Kustra R, Chan AK, Hamilton MA, et al. Individual and social determinants of SARS-CoV-2 testing and positivity in Ontario, Canada: a

population-wide study. Can Med Assoc J [Internet]. 2021 May 17 [cited 2024 May 30];193(20):E723–34. Available from: http://www.cmaj.ca/lookup/doi/10.1503/cmaj.202608

10. Public Health Agency of Canada. Canadian COVID-19 vaccination coverage report [Internet]. Ottawa: Public Health Agency of Canada; 2024 [cited 2024 May 30]. Available from: https://health-infobase.canada.ca/covid-19/vaccination-coverage/

11. Colwill K, Galipeau Y, Stuible M, Gervais C, Arnold C, Rathod B, et al. A scalable serology solution for profiling humoral immune responses to SARS-CoV-2 infection and vaccination. Clin Transl Immunol [Internet]. 2022 Jan [cited 2024 May 30];11(3):e1380. Available from: https://onlinelibrary.wiley.com/doi/10.1002/cti2.1380

12. Breznik JA, Zhang A, Huynh A, Miller MS, Nazy I, Bowdish DME, et al. Antibody Responses 3-5 Months Post-Vaccination with mRNA-1273 or BNT163b2 in Nursing Home Residents. J Am Med Dir Assoc [Internet]. 2021 Dec [cited 2024 May 30];22(12):2512–4. Available from: https://linkinghub.elsevier.com/retrieve/pii/S1525861021009026

13. Murphy TJ, Swail H, Jain J, Anderson M, Awadalla P, Behl L, et al. The evolution of SARS-CoV-2 seroprevalence in Canada: a time-series study, 2020–2023. Can Med Assoc J [Internet]. 2023 Aug 14 [cited 2024 May 30];195(31):E1030–7. Available from: http://www.cmaj.ca/lookup/doi/10.1503/cmaj.230249

14. Boucher E, Cao C, D’Mello S, Duarte N, Donnici C, Duarte N, et al. Occupation and SARS-CoV-2 seroprevalence studies: a systematic review. BMJ Open [Internet]. 2023 Feb [cited 2024 May 30];13(2):e063771. Available from: https://bmjopen.bmj.com/lookup/doi/10.1136/bmjopen-2022-063771

15. Kronfli N, Dussault C, Maheu-Giroux M, Halavrezos A, Chalifoux S, Park H, et al. Importance of occupation for SARS-CoV-2 seroprevalence and COVID-19 vaccination among correctional workers in Quebec, Canada: A cross-sectional study. Front Public Health [Internet]. 2022 Nov 9 [cited 2024 May 30];10:1021871. Available from: https://www.frontiersin.org/articles/10.3389/fpubh.2022.1021871/full

16. Brousseau N, Morin L, Ouakki M, Savard P, Quach C, Longtin Y, et al. SARS-CoV-2 seroprevalence in health care workers from 10 hospitals in Quebec, Canada: a cross-sectional study. Can Med Assoc J [Internet]. 2021 Dec 13 [cited 2024 Feb 23];193(49):E1868–77. Available from: http://www.cmaj.ca/lookup/doi/10.1503/cmaj.202783

17. Andrew MK, Godin J, LeBlanc J, Boivin G, Valiquette L, McGeer A, et al. Older Age and Frailty are Associated with Higher Mortality but Lower ICU Admission with COVID-19. Can Geriatr J [Internet]. 2022 Jun 1 [cited 2024 May 30];25(2):183–96. Available from: https://cgjonline.ca/index.php/cgj/article/view/546

18. Brockman MA, Mwimanzi F, Lapointe HR, Sang Y, Agafitei O, Cheung PK, et al. Reduced Magnitude and Durability of Humoral Immune Responses to COVID-19 mRNA Vaccines Among Older Adults. J Infect Dis [Internet]. 2022 Apr 1 [cited 2024 May 30];225(7):1129–40. Available from: https://academic.oup.com/jid/article/225/7/1129/6458430

19. Breznik JA, Rahim A, Zhang A, Ang J, Stacey HD, Bhakta H, et al. Early Omicron infection is associated with increased reinfection risk in older adults in long-term care and retirement facilities. eClinicalMedicine [Internet]. 2023 Sep [cited 2024 May 30];63:102148. Available from: https://linkinghub.elsevier.com/retrieve/pii/S2589537023003255

20. Statistics Canada. Sex differences in COVID-19 deaths in the early months of the pandemic in Canada: An examination with an immigration lens. 2023 [cited 2024 May 30]; Available from: https://www150.statcan.gc.ca/n1/pub/82-003-x/2023011/article/00001-eng.htm

21. Van Ingen T, Brown KA, Buchan SA, Akingbola S, Daneman N, Warren CM, et al. Neighbourhood-level socio-demographic characteristics and risk of COVID-19 incidence and mortality in Ontario, Canada: A population-based study. Varga C, editor. PLOS ONE [Internet]. 2022 Oct 20 [cited 2024 May 30];17(10):e0276507. Available from: https://dx.plos.org/10.1371/journal.pone.0276507

22. Khanijahani A, Iezadi S, Gholipour K, Azami-Aghdash S, Naghibi D. A systematic review of racial/ethnic and socioeconomic disparities in COVID-19. Int J Equity Health [Internet]. 2021 Nov 24 [cited 2024 May 30];20(1):248. Available from: https://equityhealthj.biomedcentral.com/articles/10.1186/s12939-021-01582-4

23. Udell JA, Behrouzi B, Sivaswamy A, Chu A, Ferreira-Legere LE, Fang J, et al. Clinical risk, sociodemographic factors, and SARS-CoV-2 infection over time in Ontario, Canada. Sci Rep [Internet]. 2022 Jun 24 [cited 2024 May 30];12(1):10534. Available from: https://www.nature.com/articles/s41598-022-13598-z

24. Schellenberg G, Fonberg J. Housing characteristics and staying at home during the COVID-19 pandemic [Internet]. Statistics Canada; 2020 [cited 2024 May 30]. Available from: https://www150.statcan.gc.ca/n1/pub/45-28-0001/2020001/article/00009-eng.htm

25. Yang FJ, Aitken N. People living in apartments and larger households were at higher risk of dying from COVID-19 during the first wave of the pandemic [Internet]. Statistics Canada; 2021 [cited 2024 May 30]. Available from: https://www150.statcan.gc.ca/n1/pub/45-28-0001/2021001/article/00004-eng.htm

26. 2016 Census of Population [Internet]. Statistics Canada; 2021. Available from: https://www12.statcan.gc.ca/census-recensement/2016/index-eng.cfm

27. Brown KA, Stall NM, Joh E, Allen U, Bogoch II, Buchan SA, et al. COVID-19 Vaccination Strategy for Ontario Using Age and Neighbourhood-Based Prioritization [Internet]. Ontario COVID-19 Science Advisory Table; 2021 Feb [cited 2024 May 30]. Available from: https://covid19-sciencetable.ca/sciencebrief/covid-19-vaccination-strategy-for-ontario-using-age-and-neighbourhood-based-prioritization

28. Mishra S, Stall NM, Ma H, Odutayo A, Kwong JC, Allen U, et al. A Vaccination Strategy for Ontario COVID-19 Hotspots and Essential Workers [Internet]. Ontario COVID-19 Science Advisory Table; 2021 Apr [cited 2024 May 30]. Available from: https://covid19-sciencetable.ca/sciencebrief/a-vaccination-strategy-for-ontario-covid-19-hotspots-and-essential-workers

29. Brophy J, Keith M, Hurley M. Breaking Point: Violence Against Long-Term Care Staff. NEW Solut J Environ Occup Health Policy [Internet]. 2019 May [cited 2024 May 30];29(1):10–35. Available from: http://journals.sagepub.com/doi/10.1177/1048291118824872

30. Syed IU. Racism, racialization, and health equity in Canadian residential long term care: A case study in Toronto. Soc Sci Med [Internet]. 2020 Nov [cited 2024 May 30];265:113524. Available from: https://linkinghub.elsevier.com/retrieve/pii/S0277953620307437

31. Pichler S, Wen K, Ziebarth NR. COVID-19 Emergency Sick Leave Has Helped Flatten The Curve In The United States: Study examines the impact of emergency sick leave on the spread of COVID-19. Health Aff (Millwood) [Internet]. 2020 Dec 1 [cited 2024 May 30];39(12):2197–204. Available from: http://www.healthaffairs.org/doi/10.1377/hlthaff.2020.00863

32. Ma H, Yiu KCY, Baral SD, Fahim C, Moloney G, Darvin D, et al. COVID-19 Cases Among Congregate Care Facility Staff by Neighborhood of Residence and Social and Structural Determinants: Observational Study. JMIR Public Health Surveill [Internet]. 2022 Oct 4 [cited 2024 May 30];8(10):e34927. Available from: https://publichealth.jmir.org/2022/10/e34927

33. Shen K. Relationship between nursing home COVID-19 outbreaks and staff neighborhood characteristics. Bayer A, editor. PLOS ONE [Internet]. 2022 Apr 19 [cited 2024 May 30];17(4):e0267377. Available from: https://dx.plos.org/10.1371/journal.pone.0267377

34. Bechini A, Lorini C, Zanobini P, Mandò Tacconi F, Boccalini S, Grazzini M, et al. Utility of Healthcare System-Based Interventions in Improving the Uptake of Influenza Vaccination in Healthcare Workers at Long-Term Care Facilities: A Systematic Review. Vaccines [Internet]. 2020 Apr 5 [cited 2024 May 30];8(2):165. Available from: https://www.mdpi.com/2076-393X/8/2/165

Abbreviations

CI	Confidence Interval
DBS	Dried Blood Spot
ELISA	Enzyme-linked immunoassay
LTCH	Long Term Care Homes
RH	Retirement Homes
PR	Prevalence ratio (age-adjusted)

Click here to access/download
Supporting Information
Seroprevalence appendices_v2_Feb1624.docx

Supporting Information 2

Click here to access/download
Supporting Information
Additional file 2.docx

Ethics approval (updated)

Click here to access/download

Supporting Information

20-347_REB Approvals 2021-2024.pdf

1. Please complete all items on the Human Participants Research Checklist that are relevant for your submission, by following this link: http://journals.plos.org/plosone/s/file?id=dc11/PLOSOne_Human_Subjects_Research_Checklist.docx. There may be overlap between the checklist items and other queries listed here; please address any duplicated queries both in your response email and on the checklist itself. Upload the completed Human Participants Research checklist as file type "Other" when you re-submit your manuscript. This document is for internal journal use only and will not be published if your article is accepted. The requested information will help us to assess whether your submission complies with PLOS's policies and adheres to applicable reporting standards. Note that your manuscript may be rejected if you provide incomplete or inadequate responses to the checklist questions and that changing the 'Section/Category' of your article does not affect this requirement.

Thank you. We have completed the checklist and we have uploaded our approved REB letter as an additional file.

2. Your study includes live participants. Please include an Ethics Statement subsection to your Materials and Methods, to include:
A statement that formal consent was obtained (including whether it was verbal or written) OR the reason consent was not obtained (e.g. anonymity)

Thank you this has been added to the methods. All participants provided written consent via paper-copy or online survey form.

Thank you for your consideration of our manuscript
