# Supplementary material for: Patterns of SARS-CoV-2 seropositivity among essential workers in long term care and retirement homes in Ontario, Canada: A descriptive cross-sectional study
Source: PLOS Glob Public Health. 2025 Mar 28;5(3):e0004294. doi: 10.1371/journal.pgph.0004294 (PMC11952236; doi:10.1371/journal.pgph.0004294)
Supplement: Attachment — Submitted filename: Response to reviewers.docx [file pgph.0004294.s007.docx]

1. Please complete all items on the Human Participants Research Checklist that are relevant for your submission, by following this link: <http://journals.plos.org/plosone/s/file?id=dc11/PLOSOne_Human_Subjects_Research_Checklist.docx>. There may be overlap between the checklist items and other queries listed here; please address any duplicated queries both in your response email and on the checklist itself. Upload the completed Human Participants Research checklist as file type “Other” when you re-submit your manuscript. This document is for internal journal use only and will not be published if your article is accepted. The requested information will help us to assess whether your submission complies with PLOS’s policies and adheres to applicable reporting standards. Note that your manuscript may be rejected if you provide incomplete or inadequate responses to the checklist questions and that changing the ‘Section/Category’ of your article does not affect this requirement.

Thank you. We have completed the checklist and we have uploaded our approved REB letter as an additional file.

2. Your study includes live participants. Please include an Ethics Statement subsection to your Materials and Methods, to include:

A statement that formal consent was obtained (including whether it was verbal or written) OR the reason consent was not obtained (e.g. anonymity)

Thank you this has been added to the methods. All participants provided written consent via paper-copy or online survey form.

Thank you for your consideration of our manuscript
