# Supplementary material for: Patterns of SARS-CoV-2 seropositivity among essential workers in long term care and retirement homes in Ontario, Canada: A descriptive cross-sectional study
Source: PLOS Glob Public Health. 2025 Mar 28;5(3):e0004294. doi: 10.1371/journal.pgph.0004294 (PMC11952236; doi:10.1371/journal.pgph.0004294)
Supplement: Attachment — Submitted filename: Response to Editor.docx [file pgph.0004294.s009.docx]

Dear Dr. Fahim,

We've checked your submission and before we can proceed, we need you to address the following issues:

1. We note that you have indicated that there are restrictions to data sharing for this study. For studies involving human research participant data or other sensitive data, we encourage authors to share de-identified or anonymized data. However, when data cannot be publicly shared for ethical reasons, we allow authors to make their data sets available upon request. For information on unacceptable data access restrictions, please see http://journals.plos.org/plosone/s/data-availability#loc-unacceptable-data-access-restrictions.

Before we proceed with your manuscript, please address the following prompts:

a) If there are ethical or legal restrictions on sharing a de-identified data set, please explain them in detail (e.g., data contain potentially identifying or sensitive patient information, data are owned by a third-party organization, etc.) and who has imposed them (e.g., a Research Ethics Committee or Institutional Review Board, etc.). Please also provide contact information for a data access committee, ethics committee, or other institutional body to which data requests may be sent.

b) If there are no restrictions, please upload the minimal anonymized data set necessary to replicate your study findings to a stable, public repository and provide us with the relevant URLs, DOIs, or accession numbers. Please see http://www.bmj.com/content/340/bmj.c181.long for guidelines on how to de-identify and prepare clinical data for publication. For a list of recommended repositories, please see https://journals.plos.org/plosone/s/recommended-repositories. You also have the option of uploading the data as Supporting Information files, but we would recommend depositing data directly to a data repository if possible.

**🡪 Thank you, we have revisited the issue with our research ethics board. The REB chair has noted that a de-identified data set to be shared has been approved. In keeping with REB requirements, we have collapsed any cells with fewer than n=5 to ensure that the data do not lead to participant identification.
The data have now been made available at:** [**OSF | Wellness Hub: Understanding COVID-19 Transmission through Implementing and Evaluating an Intervention to Support Wellness, IPAC, Vaccine Uptake, and other Wraparound Care Needs in LTCH/RHs.**](https://osf.io/pmur4/)

Please update your Data Availability statement in the submission form accordingly.

- Thank you, we have updated to the data availability statement as follows. “The study data used in this manuscript have been de-identified and made available via Open Science Framework. Data can be accessed at: <https://osf.io/zt7ux> “
